# New Stable Gallium(III) and Indium(III) Complexes with Thiosemicarbazone Ligands: A Biological Evaluation

**DOI:** 10.3390/molecules29020497

**Published:** 2024-01-19

**Authors:** Lorenzo Verderi, Mirco Scaccaglia, Martina Rega, Cristina Bacci, Silvana Pinelli, Giorgio Pelosi, Franco Bisceglie

**Affiliations:** 1 Department of Chemistry, Life Sciences and Environmental Sustainability, University of Parma, 43124 Parma, Italy; lorenzo.verderi@unipr.it (L.V.); mirco.scaccaglia@unipr.it (M.S.); giorgio.pelosi@unipr.it (G.P.); 2 Department of Veterinary Science, University of Parma, Strada del Taglio 10, 43126 Parma, Italy; martina.rega@unipr.it (M.R.); cristina.bacci@unipr.it (C.B.); 3 Department of Medicine and Surgery, University of Parma, Via Gramsci 14, 43126 Parma, Italy; silvana.pinelli@unipr.it; 4 Centre of Excellence for Toxicological Research (CERT), University of Parma, 43124 Parma, Italy

**Keywords:** thiosemicarbazone, gallium(III), indium(III), antitumour, antibiotic

## Abstract

The aim of this work is to explore a new library of coordination compounds for medicinal applications. Gallium is known for its various applications in this field. Presently, indium is not particularly important in medicine, but it shares a lot of chemical traits with its above-mentioned lighter companion, gallium, and is also used in radio imaging. These metals are combined with thiosemicarbazones, ligating compounds increasingly known for their biological and pharmaceutical applications. In particular, the few ligands chosen to interact with these hard metal ions share the ideal affinity for a high charge density. Therefore, in this work we describe the synthesis and the characterization of the resulting coordination compounds. The yields of the reactions vary from a minimum of 21% to a maximum of 82%, using a fast and easy procedure. Nuclear Magnetic Resonance (NMR) and Infra Red (IR) spectroscopy, mass spectrometry, elemental analysis, and X-ray Diffraction (XRD) confirm the formation of stable compounds in all cases and a ligand-to-metal 2:1 stoichiometry with both cations. In addition, we further investigated their chemical and biological characteristics, via UV-visible titrations, stability tests, and cytotoxicity and antibiotic assays. The results confirm a strong stability in all explored conditions, which suggests that these compounds are more suitable for radio imaging applications rather than for antitumoral or antimicrobic ones.

## 1. Introduction

Biomedicinal uses of gallium are not new [1]: its applications spread from antitumor drugs [2,3,4], to antibiotics [5,6,7] and to radio imaging too [8,9,10,11]. Its activity takes place via different mechanisms depending on the application. Most of Ga(III) antitumoural or antibiotic activity derives from its similarity to Fe(III) ion [12], which allows it to enter iron metabolic pathways and break them down. On the other hand, gallium activity in radio imaging as ^68^Ga(III) and ^67^Ga(III) is based on long-range radiations (in the first case positron emissions, in the latter γ-rays), not requiring direct interaction with the environment: in fact, the ion itself would cause heavy drawbacks to the patient, exactly because of the toxicity mechanisms mentioned before. To this end, when employed in therapeutics, Ga(III) usually needs to be released in the biological environment at a certain time (even if usually a chelator is necessary to tune its efficacy and selectivity), whereas an unbreakable coordination system is required for radio imaging to minimize toxicity and to convey the radioactive source in the metabolism.

Indium is mostly known for its applications in material science; on the contrary, its use in biochemistry and pharmaceuticals is very limited compared to gallium. It is used in radio imaging as ^111^In [13,14,15,16] and ^113m^In [17], and few In(III) compounds have shown antimicrobial and cytotoxic efficacy [18]. Anyway, its chemical similarity to gallium and its enhanced metallic character constitutes a good start to investigate its coordination chemistry and eventual biomedicinal activity.

Research on innovative coordination compounds of Ga(III) and In(III) then appears interesting, and thiosemicarbazones were chosen as target ligands for different reasons. Thiosemicarbazones (>C=N–NH–C(S)–N<) are generally good chelators for different metal ions, especially thanks to their mixed hard/soft character [19,20]. Heterocyclic-based thiosemicarbazones, in particular, play a pivotal role in biological systems due to their diverse pharmacological activities. These compounds exhibit promising antimicrobial, antiviral, and anticancer properties, making them valuable candidates for drug development. Their ability to coordinate with metal ions further enhances their biological relevance, influencing enzymatic processes and cellular functions. The unique structural features of heterocyclic thiosemicarbazones contribute to their therapeutic potential, showcasing their significance in the pursuit of novel pharmaceutical agents [21]. In addition, they display a variety of activities both by themselves [19] and within coordination compounds of a vast range of metals [2,6,20,22,23,24,25,26,27,28,29,30], which justify their increasing importance in biochemistry and pharmaceutics (see Figure S1).

The majority of the reported thiosemicarbazone complexes involve soft/intermediate metals, whereas Ga(III) and In(III) are considered hard. Nonetheless, some ligands presented in a recent paper of ours [27] show instead a clear “hardened” character due to the presence of a hydroxyl group, making them good candidates for a possible Ga(III) and In(III) coordination.

The stability of metal complexes plays a pivotal role in influencing their biological activity. In biological systems, metal ions often form complexes with various ligands, including proteins, enzymes, and other biomolecules. The stability of these metal complexes is crucial for their functionality and reactivity within biological processes. Changes in the coordination environment, such as ligand exchange or dissociation of the metal from the complex, can significantly impact the type of biological activity of these complexes. Understanding and controlling the stability of metal complexes are, therefore, key factors in elucidating their roles in biological systems and developing applications in fields such as medicine and catalysis.

**Figure 1:**
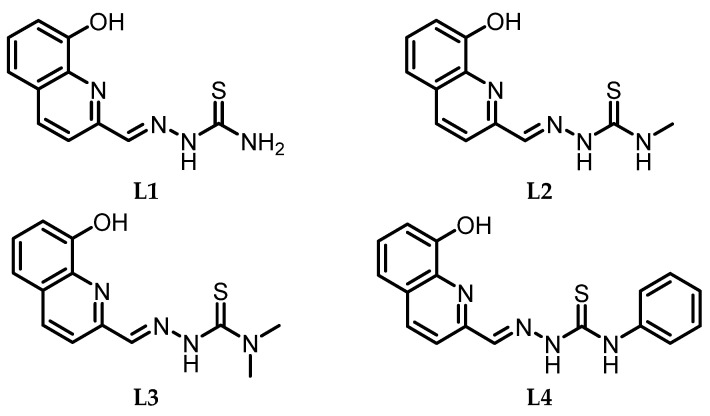
Chosen thiosemicarbazonic ligands for Ga(III) and In(III) coordination, previously described [27].

To this end, this work focused on the synthesis and full characterization of a whole set of innovative coordination compounds of gallium and indium with four different thiosemicarbazone ligands (see Figure 1), other than biological assays to investigate their properties in relation to their stability.

## 2. Results and Discussion

### 2.1. Synthesis

Ligand formation by Schiff-base condensation was previously optimized [27], and the conditions are reported anyway inside the present work (for further details, see Section 3).

Complexes synthesis (see Section 3) was carried out in an alcoholic solvent with an acidic catalyst following slightly different procedures (see Table 1), starting from the nitrate salts of the relative cations. In general, this synthesis shows a series of advantages: it took a relatively short time (a few hours maximum), employed a low quantity of alcohol as a solvent, returning products as pure precipitates, with no need for long and expensive purification steps such as column chromatography and recrystallization. Yields varied from 21% up to 82% depending on the complex. All the compounds were characterized via Elemental Analysis (EA), ^1^H NMR, ^13^C NMR and IR spectroscopy, and Electrospray Ionization-Mass Spectrometry (ESI-MS) (for further details, see Section 3 or Supplementary Materials). The target compounds were then obtained successfully (see Table 1) and their properties were further investigated thereafter.

### 2.2. Structural Information

**Figure 2:**
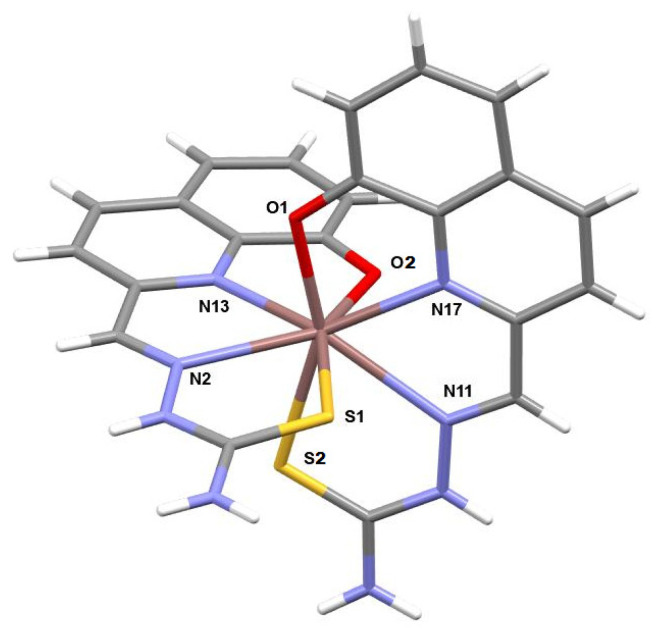
Representation of the cationic moiety of **In1** complex.

The crystal structure reveals a peculiar coordination around the In^3+^ ion where two anionic ligand molecules behave as quadridentate and are perpendicular (89.51°) one with respect to the other (see Figure 2 *and* Figure S4).

Looking at the coordination bonds it is apparent that the distance between the In^3+^ ion and the imine nitrogens (2.563(1) and 2.537(1) Å) are remarkably longer that the other two In-N bonds (2.329(1) and 2.321(1) Å) and this is probably due to the stronger interaction of a hard In^3+^ ion with the two negatively charged oxygen atoms (2.223(1) and 2.253(1) Å) (Table 2).

**Figure 3:**
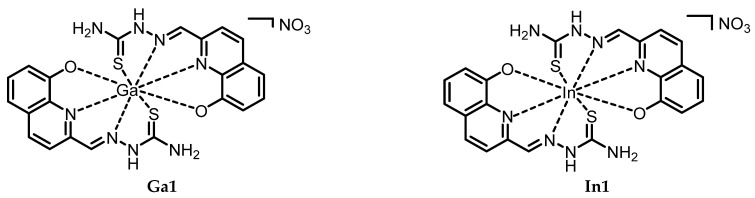

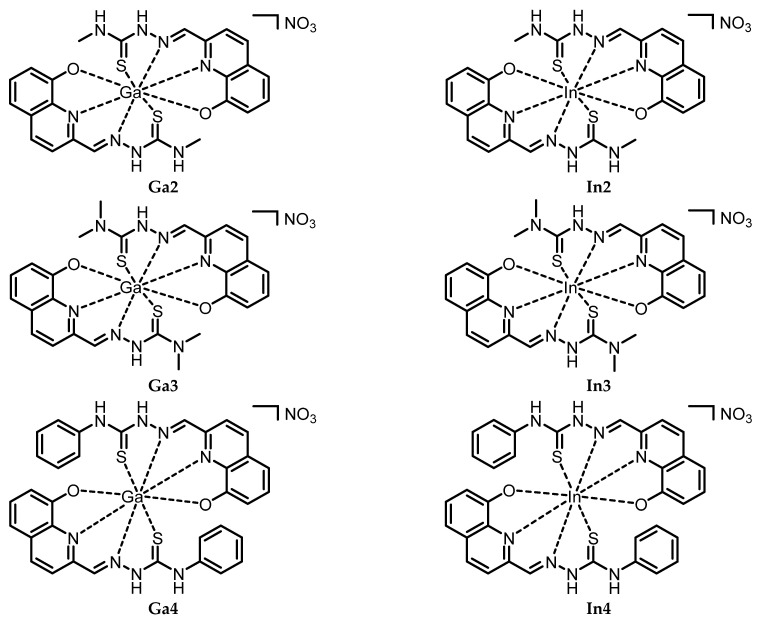
Plausible structure of the synthesized compounds, according to characterization and **In1** XRD information.

The packing is determined by the presence, besides the nitrate ion necessary to compensate for the positive charge of the metal complex moiety, of three molecules of ethanol, and one of water which determine a complex network of hydrogen bonds (Figure S3). The stoichiometry and coordination system found in **In1** has been exploited as a model for the whole set of compounds (see Figure 3). The phenolic oxygen seems to have a strong affinity for the cation since it is deprotonated before coordination, while the thiosemicarbazonic NH group (which is known to usually be deprotonated before complexations to give a large S^−^ group interacting with soft metals) did not, demonstrating that these cations have a stronger affinity with oxygen rather than with sulfur.

In addition, a large coordination number (8) is shown, involving most of the heteroatoms of the ligands in the interaction with the metal.

### 2.3. UV-Visible Titrations

The formation of the coordination compounds was investigated via UV-visible titrations in a Phosphate Buffered Saline (PBS) buffer with 5% Dimethyl Sulfoxide (DMSO), which is an accepted mimic of an environment with human physiological pH. Ligands concentration was 40 μM for **Ga1**–**Ga4**, **In1,** and **In2**, whereas it was 20 μM for **In3** and **In4**. Titrant solution containing the metal nitrates was 400 μM concentrated for **Ga1**–**Ga4**, **In1,** and **In2**, whereas they were 200 μM for **In3** and **In4**. Additions were executed using micropipettes sensible to 10 μL in a progressive manner: starting with 20 μL for the first 10 additions, up to 40 μL and 100 μL additions at the end of each titration.

**Figure 4:**
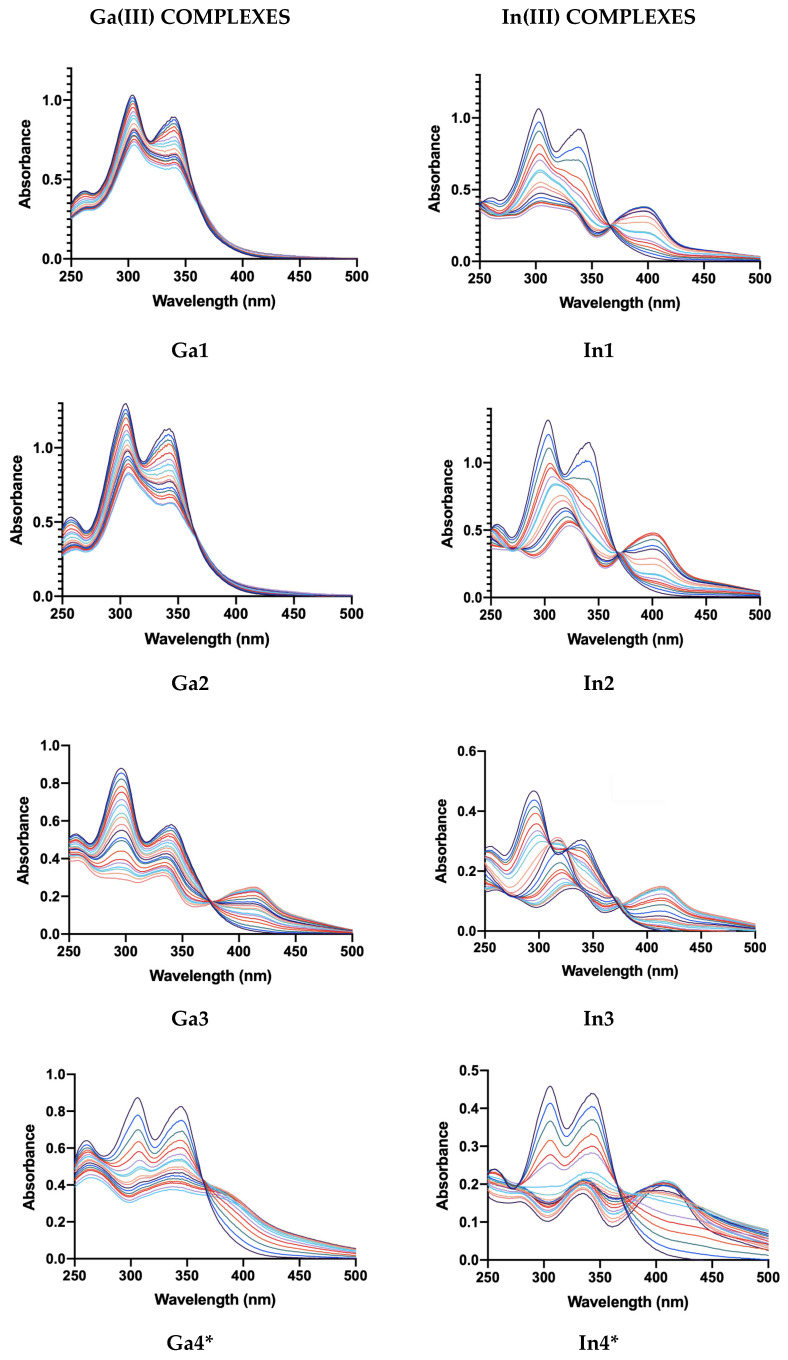
Titration profiles of the synthesized coordination compounds (* the baseline raised through the titration due to slight formation of precipitate).

The titration profiles (see Figure 4) indicate the formation of every complex: most of the indium compounds, and the last two gallium as well, display evident changes along with metal concentration increase, such as evident isosbestic point and a new absorption band around 400 nm; on the other hand, **Ga1** and **Ga2** show analog traits but in a less evident manner. Both ways, the absorption band emerging at 400 nm seems to indicate stable coordination in every case and is probably relative to a Metal-to-Ligand Charge Transfer (MLCT) since both the ions have a d^10^ configuration and are coordinated to electron-withdrawing systems such as the hydroxy-quinolines, which presents available π* orbitals that are best to host electron density coming from metal back-donation. From the stability point of view, these titration profiles are a qualitative indication of the strong affinity between these ligands and metals in a pseudo-physiological medium at 25 °C and atmospheric pressure.

### 2.4. Stability Assay

The coordination compounds were dissolved in DMSO and the stability assays were performed in a PBS buffer with 2% of DMSO. Electronic spectra were taken at the UV-visible spectrophotometer before and after an incubation at atmospheric pressure and at 37 °C for 24 h.

**Figure 5:**
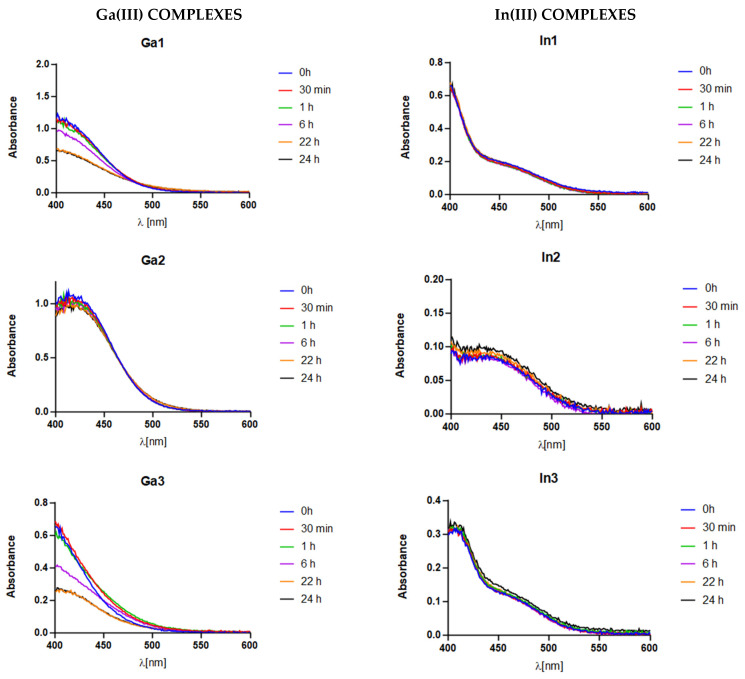

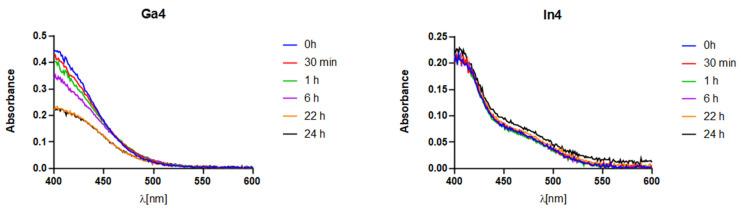
Stability assays of the coordination compounds.

All the solutions showed to be overall stable along a 24 h interval (see Figure 5). The absorbance values were slightly lower in certain cases due to a minimal precipitation of the compounds, but most of all no qualitative changes are reported. This indicates that the complexes are stable, therefore, no metal ions were released in the solution. This informs about the strong affinity between these metals and ligands in an in vivo like environment.

### 2.5. Cytotoxicity Assay

In order to investigate the properties of these compounds, ligands, and the relative complexes were tested in vitro on human cancer cells belonging to lung cancer cell line A549. As shown in Table S1 (see Supplementary Materials), cellular vitality is scarcely inhibited by any coordination compound. It is possible to infer that, since both the metals and the ligands are reported as cytotoxic by themselves, the interaction between the cations and the thiosemicarbazones is too stable even to have an activity inside human cells. It is necessary to specify that the comparison between **L3** and its coordination compounds is exceptionally different since the ligand has a higher IC_50_ (71 µM ± 1) than both the gallium and indium complexes (50 µM ± 1 and 65 µM ± 2, respectively). Anyways, it is useful to notice that those values are invariably high for a hypothetic therapeutic application.

### 2.6. Antibiotic Assay

The antibiotic activity was assessed by determining the minimum inhibitory concentrations (MIC) of the compounds against two distinct bacterial strains: *Escherichia coli* and *MRSA* (Methicillin-Resistant *Staphylococcus aureus*). This analysis allowed us to compare a Gram-negative strain (*E. coli*) and a Gram-positive one methicillin-resistant *Staphylococcus aureus* (*MRSA*): these two types of bacteria display different grades of vulnerability to antimicrobials, even if, in general, drugs share more effectiveness against Gram-positive bacteria; this comparison widens the information spectrum about the activity of these compounds.

As it happened with cancer cells, these compounds seem to have very low activity on bacteria, testifying once again to their great stability in vitro (see Table 3). It is once again interesting to notice the same exception seen with the cytotoxicity assay: **L3** MIC for *MRSA* is higher than **Ga3** one, addressing a mild toxicity of this coordination compound in some conditions. On the other hand, **In3** follows the general scheme with a higher MIC value than **L3**.

## 3. Materials and Methods

All common laboratory chemicals were purchased from commercial sources and used without further purification: 8-hydroxy-2-quinolinecarboxaldehyde, ≥96% (Sigma-Aldrich, St. Louis, MO, USA); thiosemicarbazide, ≥99.9% (Fluka, Buchs, Switzerland); 4-methyl-3-thiosemicarbazide, 97% (Fluorochem, Hadfield, UK); 4,4-dimethyl-3-thiosemicarbazide, ≥98.0% (TCI, Tokyo, Japan); 4-phenyl-3-thiosemicarbazide, 99% (Sigma-Aldrich); gallium nitrate hydrate, 99.9% (Sigma-Aldrich); indium nitrate hydrate, 99.9% (Sigma-Aldrich). NMR was recorded on a Bruker Anova spectrometer at 400 MHz (Billerica, MA, USA), with chemical shift reported in δ units (ppm). NMR spectra were referenced relative to residual NMR solvent peaks. The solvent used in the spectra’s acquisitions is DMSO-*d*_6_. The FT-IR measurements were recorded on Nicolet 5PC FT-IR (Rodano, MI, Italy) in the 4000–400 cm^−1^ range, equipped with the ATR accessory. Elemental analyses were performed using the Thermofisher Scientific Flashsmart CHNS Elemental Analyzer (Rodano, MI, Italy). ESI-MS were recorded on a Waters Acquity Ultraperformance ESI-MS spectrometer with a Single Quadrupole Detector (Sesto San Giovanni, MI, Italy). UV/Vis spectra were collected using a Thermofisher Scientific Evolution 260 Bio Spectrophotometer (Rodano, MI, Italy), using quartz cuvettes of 1 cm path length.

### 3.1. Preparation of the Complexes

The ligand preparation is reported in the Supplementary Materials.

**Ga1.** (((8-Hydroxyquinolin-2-yl)methylidene)amino)thiourea (**L1**; 51 mg, 0.21 mmol) was dissolved in MeOH (10 mL) at reflux. A solution of hydrated Ga(NO_3_)_3_ (52 mg, 0.20 mmol) in 3 mL in MeOH was then added dropwise to the ligand mixture and left stirring for 2 h at RT. A turbid dark yellow mixture was then obtained and cooled down to 0 °C in an ice bath. The yellow product was then separated via filtration (65%). Anal. Calcd. for C_44_H_38_Ga_2_N_10_O_11_S_4_ (2**Ga1** + H_2_O): C, 41.86; N, 19.97; H, 3.03; S, 10.16. Exp.: C, 42.38; N, 19.21; H, 2.90; S, 9.42. IR (ATR, cm^−1^): 3418, 3228, 3139, 3076, and 2935 w (N-H, O-H, and C-H), 1526 s (C=N), 1385 m and 745 s (C=S). ESI-MS *m*/*z* (%): 313.5 ([Ga + L − 2H]^+^ 71.1), 558.6 ([Ga + 2L − 2H]^+^ 100.0). ^1^H-NMR (400 MHz, DMSO-*d*_6_): [ppm] 12.24 (s, 1H, N**H**), 8.86 (br, 1H, N=C-**H**), 8.48 (br, 1H, C**H** ar.), 8.44 (d, 1H, C**H** ar.), 8.32 (br, 1H, C**H** ar.), 8.29 (t, 1H, C**H** ar.), 7.52 (s, 1H, N**H**), 7.38 (d, 1H, C**H** ar.), 7.19 (s, 1H, N**H**). ^13^C-NMR (400 MHz, DMSO-*d*_6_): [ppm] 178.4 (**C**=N aliph.), 155.2 (**C**=S), 153.4 (**C**-OH), 151.9–150.5 (**C**=N ar.), 142.4 (C=**C**=N ar.), 139.3–138.8–138.1 (C=**C**=C ar.), 136.1 (C=**C**H-C ar., N-para), 130.0–128.8 (C=**C**H-C ar., OH-para), 128.4–128.1 (C=**C**H-C ar., N-meta), 120.0–118.4–117.8 (C=**C**H-C ar., OH-para), 114.1–113.5–112.1 (C=**C**H-C ar., OH-ortho).
**In1.** (((8-Hydroxyquinolin-2-yl)methylidene)amino)thiourea (**L1**; 49 mg, 0.20 mmol) was dissolved in MeOH (10 mL) at reflux. A solution of hydrated In(NO_3_)_3_ (61 mg, 0.20 mmol) in 3 mL in MeOH was then added dropwise to the ligand mixture and left stirring for 2 h at RT. A clear dark red solution was then obtained and cooled down to 0 °C in an ice bath. The solvent was removed via rotary evaporation and the resulting dry solid was recovered with EtOH (10 mL). The solution was left at 0 °C overnight, and the product precipitated. After filtration, the product was washed with diethyl ether, to yield a red solid (21%). Anal. Calcd for C_22_H_30_InN_9_O_11_S_2_ (**In1** + 6H_2_O): C, 34.07; N, 16.90; H, 3.90; S, 8.27. Exp.: C, 33.55; N, 17.16; H, 2.45; S, 7.76. IR (ATR, cm^−1^): 3451, 3286, 3186, and 3062 w (N-H, O-H, and C-H), 1575 m (C=N), 1385 s and 756 s (C=S). ESI-MS *m*/*z* (%): 359.2 ([In + L − 2H]^+^, 72.4), 605.2 ([In + 2L − 2H]^+^, 100.0). ^1^H-NMR (400 MHz, DMSO-*d*_6_): [ppm] 11.94 (s, 1H, HC=N-N**H**), 8.62 (d, 1H, C**H** ar.), 8.54 (s, 1H, N**H**), 8.52–8.33 (m, 5H, C**H** ar., +N**H**), 8.31 (s, 1H, N=C-**H**), 7.90 (d, 1H, C**H** ar.), 7.53–7.38 (m, 3H, C**H** ar.), 7.17–7.04 (m, 2H, C**H** ar.), 6.86 (d, 1H, C**H** ar.). ^13^C-NMR (400 MHz, DMSO-*d*_6_): [ppm] 178.5 (**C**=N aliph.), 158.9 (**C**=S), 152.8 (**C**-OH), 151.6 (**C**=N ar.), 141.3–140.8 (C=**C**=N ar.), 137.1 (C=**C**=C ar.), 136.3 (C=**C**H-C ar., N-para), 131.4 (C=**C**H-C ar., OH-para), 128.9–128.4 (C=**C**H-C ar., N-meta), 121.6–118.6–117.9 (C=**C**H-C ar., OH-para), 112.6–112.2–110.7 (C=**C**H-C ar., OH-ortho). XRD: CCDC No 2311577.
**Ga2.** 1-(((8-Hydroxyquinolin-2-yl)methylidene)amino)-3-methylthiourea (**L2**; 49 mg, 0.19 mmol) was dissolved in MeOH (10 mL) at reflux. A solution of hydrated Ga(NO_3_)_3_ (48 mg, 0.19 mmol) in 3 mL in MeOH was then added dropwise to the ligand mixture and left stirring for 2 h at RT. A turbid dark yellow mixture was then obtained and cooled down to 0 °C in an ice bath. The yellow product is then separated via filtration (82%). Anal. Calcd for C_48_H_50_Ga_2_N_18_O_13_S_4_ (2**Ga2** + 3H_2_O): C, 42.56; N, 18.61; H, 3.72; S, 9.47. Exp.: C, 42.58; N, 17.98; H, 3.53; S, 9.12. IR (ATR, cm^−1^): 3418, 3255, 3139, 3042 w (N-H, O-H, and C-H), 1584 (C=N), 1130 and 751 s (C=S). ESI-MS *m*/*z* (%): 587.3 ([Ga + 2L − 2H]^+^, 12.4). ^1^H-NMR (400 MHz, DMSO-*d*_6_): [ppm] 8.91 (s, 1H, N=C-**H**), 8.91 (br, 1H, C=C**H**=C N-para), 8.77 (s, 1H, N**H**), 8.66 (br, 1H, C=C**H**=C N-meta), 7.66 (t, 1H, C=C**H**=C OH-meta), 7.42 (dd, 1H, C=C**H**=C OH-ortho), 7.20 (dd, 1H, C=C**H**=C OH-para), 4.05 (s, 3H, C**H**_3_).^13^C NMR (400 MHz, DMSO-*d*_6_): [ppm] 181.7 (**C**=N aliph.), 155.8 (**C**=S), 151.2 (**C**-OH), 140.8 (**C**=N ar.), 136.6 (C=**C**=N ar.), 131.0 (C=**C**=C ar.), 129.4 (C=**C**H-C ar., N-para), 120.6 (C=**C**H-C ar., OH-para), 115.1 (C=**C**H-C ar., N-meta), 114.1 (C=**C**H-C ar., OH-para), 49.1 (C=**C**H-C ar., OH-ortho), 34.4 (**C**H_3_).
**In2.** 1-(((8-Hydroxyquinolin-2-yl)methylidene)amino)-3-methylthiourea (**L2**; 49 mg, 0.19 mmol) was dissolved in EtOH (10 mL) at reflux. A solution of hydrated In(NO_3_)_3_ (58 mg, 0.19 mmol) in 3 mL in EtOH was then added dropwise to the ligand mixture and left stirring for 2 h. A turbid red mixture was then obtained and cooled down to 0 °C in an ice bath. After filtration, the product was washed with diethyl ether, to yield a red solid (54%). Anal. Calcd. for C_24_H_30_InN_9_O_9_S_2_ (**In2** + 4H_2_O): C, 37.56; N, 16.43; H, 3.94 S, 8.35. Exp.: C, 38.07; N, 15.55; H, 3.11; S, 8.60. IR (ATR, cm^−1^): 3330 and 3167 w (N-H, O-H and C-H), 1589 m (C=N), 1138 and 742 s (C=S). ESI-MS *m*/*z* (%): 633.0 ([In + 2L − 2H]^+^, 100.0), 373.0 ([In + L − 2H]^+^, 24.7). ^1^H-NMR (400 MHz, DMSO-*d*_6_): [ppm] 9.84 (s, 1H, HC=N-N**H**), 8.91 (s, 1H, N**H**), 8.68 (s, 1H, N**H**), 8.63 (br, 1H, C**H** ar.), 8.62 (s, 1H, N-**H**), 8.56 (d, 1H, C**H** ar.), 8.32 (d, 1H, C**H** ar.), 8.02 (s, 1H, N=C-**H**), 7.98 (s, 1H, N=C-**H**), 7.87 (d, 1H, C**H** ar.), 7.48 (t, 1H, C**H** ar.), 7.45 (dd, 1H, C**H** ar.), 7.41 (dd, 1H, C**H** ar.), 7.11 (dd, 1H, C**H** ar.), 7.10 (t, 1H, C**H** ar.), 6.86 (dd, 1H, C**H** ar.), 6.77 (s, 1H, N**H**), 3.89 (s, 3H, C**H**_3_), 3.88 (s, 3H, C**H**_3_). ^13^C-NMR (400 MHz, DMSO-*d*_6_): [ppm] 182.2, 181.0 (**C**=N aliph.), 158.7 (**C**=S), 153.4 (**C**-OH), 151.7 (**C**=N ar.), 143.0, 141.1, 140.8 (C=**C**=N ar.), 138.1, 136.4 (C=**C**=C ar.), 131.0, 129.4 (C=**C**H-C ar., N-para), 128.8, 128.2, 127.5 (C=**C**H-C ar., OH-para), 121.4, 118.9 (C=**C**H-C ar., N-meta), 117.7 (C=**C**H-C ar., OH-para), 112.4, 111.9, 110.9 (C=**C**H-C ar., OH-ortho), 33.5, 32.9 (**C**H_3_).
**Ga3.** 1-(((8-Hydroxyquinolin-2-yl)methylidene)amino)-3,3-dimethylthiourea (**L3**; 40 mg, 0.14 mmol) was dissolved in MeOH (10 mL) at reflux. A solution of hydrated Ga(NO_3_)_3_ (40 mg, 0.16 mmol) in 3 mL in MeOH was then added dropwise to the ligand mixture. A clear red solution was then obtained and left stirring at RT for 2 h. The resulting red solution was then cooled down to 0 °C in an ice bath. The solvent was removed via rotary evaporation and the resulting dry solid was recovered with EtOH (10 mL). The solution was left at 0 °C overnight, and the product precipitated. After filtration, the product was washed with diethyl ether, to yield a red solid (66%). Anal. Calcd. for C_26_H_38_GaN_9_O_11_S_2_ (**Ga3** + 6H_2_O): C, 39.71; N, 16.03; H, 4.87; S, 8.15. Exp.: C, 39.74; N, 16.18; H, 3.96; S, 7.90. IR (ATR, cm^−1^): 3379, 3070 w (O-H and C-H), 1589 m (C=N), 1302 s and 729 m (C=S) ESI-MS *m*/*z* (%): 341.1 ([Ga + L − 2H]^+^, 100.0), 614.8 ([Ga + 2L − 2H]^+^, 3.8), 679.0 ([Ga + 2L + NO_3_^−^ − H]^+^, 38.8). ^1^H-NMR (400 MHz, DMSO-*d*_6_): [ppm] 8.40 (s, 1H, N=C-**H**), 8.33 (d, 1H, C**H** ar.), 8.04 (d, 1H, C**H** ar.), 7.91 (s, 1H, N-**H**), 7.45 (dd, 1H, C**H** ar.), 7.39 (t, 1H, C**H** ar.), 7.14 (dd, 1H, C**H** ar.). ^13^C NMR (400 MHz, DMSO-*d*_6_): [ppm] 181.0, 180.7 (**C**=N aliph.), 153.0 (**C**=S), 152.0 (**C**-OH), 143.2 (**C**=N ar.), 136.8, 136.0 (C=**C**=N ar.), 129.9, 128.6, 128.1 (C=**C**=C ar.), (C=**C**H-C ar., N-para), 119.1 (C=**C**H-C ar., OH-para), 117.9, 117.6 (C=**C**H-C ar., N-meta), 114.3 (C=**C**H-C ar., OH-para), 112.3 (C=**C**H-C ar., OH-ortho), 42.4 (**C**H_3_).
**In3.** 1-(((8-Hydroxyquinolin-2-yl)methylidene)amino)-3,3-dimethylthiourea (**L3**; 39 mg, 0.14 mmol) was dissolved in EtOH (10 mL) at reflux. A solution of hydrated In(NO_3_)_3_ (44 mg, 0.15 mmol) in 3 mL in EtOH was then added dropwise to the ligand mixture. A red solution was then obtained and left stirring at RT for 2 h. The red turbid mixture was then cooled down to 0 °C in an ice bath. After filtration, the product was washed with diethyl ether, to yield a red solid (47%). Anal. Calcd. for C_26_H_32_InN_9_O_8_S_2_ (**In3** + 3H_2_O): C, 40.16; N, 16.21; H, 4.15; S, 8.25. Exp.: C, 40.69; N, 15.78; H, 3.76; S, 8.50. IR (ATR, cm^−1^): 3377, 2985 and 2902 w (C-H, O-H), 1573 m (C=N), 1316 and 740 s (C=S). ESI-MS *m*/*z* (%): 387.4 ([In + L − 2H]^+^, 20.5), 661.1 ([In + 2L − 2H]^+^, 55.1). ^1^H-NMR (400 MHz, DMSO-*d*_6_): [ppm] 8.75 (s, 1H, N=C-**H**), 8.60 (d, 1H, C=C**H**=C N-para), 7.95 (d, 1H, C=C**H**=C N-meta), 7.40 (br, 1H, C=C**H**=C OH-meta), 7.40 (br, 1H, C=C**H**=C OH-ortho), 7.27 (s, 1H, N**H**), 6.79 (br, 1H, C=C**H**=C OH-para).^13^C NMR (400 MHz, DMSO-*d*_6_): [ppm] 180.7, 178.2 (**C**=N aliph.), 159.1 (**C**=S), 153.2 (**C**-OH), 152.0 (**C**=N ar.), 143.5, 140.3, 139.9 (C=**C**=N ar.), 136.5 (C=**C**=C ar.), 131.0 (C=**C**H-C ar., N-para), 128.6, 128.1 (C=**C**H-C ar., OH-para), 120.7 (C=**C**H-C ar., N-meta), 117.8, 117.6 (C=**C**H-C ar., OH-para), 112.5, 112.2, 111.6, 110.1 (C=**C**H-C ar., OH-ortho), 42.4 (**C**H_3_).
**Ga4.** 3-(((8-Hydroxyquinolin-2-yl)methylidene)amino)-1-phenylthiourea (**L4**; 51 mg, 0.16 mmol) was dissolved in MeOH (10 mL) at reflux. A solution of hydrated Ga(NO_3_)_3_ (38 mg, 0.15 mmol) in 3 mL in MeOH was then added dropwise to the ligand mixture. A clear orange solution was then obtained and left stirring at RT for 2 h. The resulting turbid orange mixture was then cooled down to 0 °C in an ice bath. After filtration, the product was washed with diethyl ether, to yield an orange solid (36%). Anal. Calcd. for C_34_H_32_GaN_9_O_8_S_2_ (**Ga4** + 3H_2_O): C, 49.29; N, 15.22; H, 3.89; S, 7.74. Exp.: C, 50.02; N, 14.68; H, 3.37; S, 7.89. IR (ATR, cm^−1^): 3288, 3123 and 2988 w (N-H, O-H and C-H), 1529 m (C=N), 1316 and 698 m (C=S). ESI-MS *m*/*z* (%): 435.3 ([Ga + L + EtOH − 2H]^+^, 100.0), 757.3 ([Ga + 2L + EtOH − 2H]^+^, 7.7). ^1^H-NMR (400 MHz, DMSO-*d*_6_): [ppm] 12.65 (s, 2H, N**H**), 10.28 (s, 2H, N**H**), 8.52 (s, 1H, N=C-**H**), 8.36 (s, 1H, N=C-**H**), 7.58–7.24 (m, 20H, C-**H** ar.). ^13^C-NMR (400 MHz, DMSO): [ppm] 177.0, 168.2 (**C**=N aliph.), 163.0, 155.6 (**C**=S), 143.2, 141.7 (**C**-OH), 139.1, 136.0, 132.7, 132.2, 129.9, 127.9, 125.9, 125.5, 120.21, 119.9, 113.9, 113.0, 109.5, 108.0, 101.8, 100.8, 98.3, 87.0, 84.1, 68.2. (**C** ar.).
**In4.** 3-(((8-Hydroxyquinolin-2-yl)methylidene)amino)-1-phenylthiourea (**L4**; 50 mg, 0.16 mmol) was dissolved in EtOH (10 mL) at reflux. A solution of hydrated In(NO_3_)_3_ (46 mg, 0.15 mmol) in 3 mL in EtOH was then added dropwise to the ligand mixture. A red solution was then obtained and left stirring at RT for 2 h. Afterwards, the solution was left for some hours at 0 °C. Diethyl ether (10 mL) was then added, and the solution was left at 0 °C for additional hours. The solvents were removed through rotary evaporation, and EtOH (3 mL) was used to recover the mixture, which was then left at 0 °C overnight, giving a red turbid mixture. After filtration, the product was washed with diethyl ether, to yield a red-orange solid (21%). Anal. Calcd. for C_34_H_38_InN_9_O_11_S_2_ (**In4** + 6H_2_O): C, 44.02; N, 13.59; H, 4.13; S, 6.91. Exp.: C, 43.56; N, 14.09; H, 3.68; S, 6.78. IR (ATR, cm^−1^): 3029 and 2775 w (N-H, O-H and C-H), 1498 m (C=N), 1311 and 693 s (C=S). ESI-MS *m*/*z* (%): 435.3 ([In + L − 2H]^+^, 4.6), 481.3 ([In + L + EtOH − 2H]^+^, 1.8), 757.3 ([In + 2L − 2H]^+^, 15.6). ^1^H-NMR (400 MHz, DMSO-*d*_6_): [ppm] 9.83 (s, 1H, N**H**), 8.68 (s, 1H, N=C**H**), 8.56 (d, 1H, C**H** ar.), 7.81 (d, 1H C**H** ar.), 7.78 (dd, 2H, C**H** ar.), 7.41 (t, 1H, C**H** ar.), 7.31 (t, 2H, C**H** ar.), 7.04 (dd, 1H, C**H** ar.), 7.03 (t, 1H, C**H** ar.), 6.81 (dd, 1H, C**H** ar.). ^13^C NMR (400 MHz, DMSO-*d*_6_): [ppm] 177.1, 175.5 (**C**=N aliph.), 159.8, 153.9 (**C**=S), 152.2, 143.4 (**C**-OH), 143.2, 140.9, 140.6, 139.5, 138.7, 137.0, 136.6, 131.5, 129.9, 129.4, 129.0, 128.8, 128.7, 126.8, 126.1, 123.2, 121.8, 121.6, 119.2, 118.3, 112.6, 112.2, 110.6 (**C** ar.).

### 3.2. UV-Visible Titrations

These experiments were carried out in a PBS buffer with 5% of DMSO. Ligands concentration was 40 μM for **Ga1**–**Ga4**, **In1,** and **In2**, whereas it was 20 μM for **In3** and **In4**. Titrant solution containing the metal nitrates in the same solvent was 400 μM concentrated for **Ga1**–**Ga4**, **In1,** and **In2**, whereas they were 200 μM for **In3** and **In4**. Additions were executed using Eppendorf™ micropipettes sensible to 10 μL in a progressive manner: we started with 20 μL for the first 10 times, up to 40 μL and 100 μL additions at the end of each titration. UV-visible spectra were taken in a 4 mL quartz cuvette 1 cm long with a a Thermofisher Scientific Evolution 260 Bio Spectrophotometer (Rodano, MI, Italy).

### 3.3. Stability Assay

The compounds were dissolved in PBS buffer with 2% DMSO, and their UV-visible spectra were taken with a Thermofisher Scientific Evolution 260 Bio Spectrophotometer (Rodano, MI, Italy), in a 700 μL quartz cuvette 1 cm long. The compounds were incubated in a standardized chamber with a temperature fixed at 37 °C at atmospheric pressure.

### 3.4. Cytotoxicity Assay

Cells were used in log-phase growth after a minimum of 24 h of adherence to culture flasks. Cells for each treatment were incubated for 24 h at different drug concentrations (0.1–100 µM). An untreated sample was used as a control. The cells were seeded into 96-well plates and incubated for 24 h, then treated with ligands and gallium and indium complexes for another 24 h. All treatments were performed in triplicate. At the fixed time-point, the MTT (3-(4,5-dimethylthiazole)-2, 5-diphenyltetrazoliumbromide) reagent was added into each well and incubated at 37 °C for 3 h. Formazan crystals were then dissolved with a 100 mL solution of HCl 0.08 M in isopropanol. The absorbance was detected with a Multiskan Ascent™ microwell plate reader equipped with a 550 nm filter (Thermo Labsystems™, Helsinki, Finland).

### 3.5. Antibiotic Assay

The samples were tested for their antimicrobial activity against *Escherichia coli* W3110 and *Staphylococcus aureus* COL (*MRSA*). Enterococcus clinical isolate strains isolated in the laboratory of the Food Inspection Unit of Parma University from the pig food chain. Resistance to vancomycin was carried by vanA gene amplification (VanA) and detected by Polymerase chain reaction (PCR). A colony of bacteria was grown in Luria–Bertani (LB) medium overnight at 37 °C. Stock solutions of 2 mM of the samples were prepared in DMSO and diluted to a starting concentration of 100 μM in Mueller Hinton (MH) medium to the starting concentration needed. For the identification of the active compound, a MIC test was set up using 96-microwells plates. The starting compounds concentration used was 100 μM and two-folds dilution were performed. Bacterial suspension in MH medium was used at a final concentration of 5 × 10^5^ CFU/mL. The total volume per each well was 150 μL including both compounds and bacterial suspension. The plates were then incubated at 37 °C for 18 h. For each assay, a control of broth only and a culture broth without antibiotics were included in two columns of the plate. Polymyxin B and Vancomycin were used as control antibiotics for *E. coli* and *MRSA*, respectively. The growth and MIC were determined by analyzing the absorbance of the bacterial suspension at 600 nm using a plate reader (Tecan instrument Infinite M1000).

## 4. Conclusions

A set of coordination compounds was successfully obtained consisting of a metal between Ga(III) and In(III), and a ligand coming from a group of four thiosemicarbazones. The synthesis we carried out displays several advantages, from the short time to the low quantity of solvent needed, giving an almost pure product without the use of expensive column chromatography.

The XRD structure of one of the compounds shows a model of the coordination system adopted by these substances, with a high coordination number, which is interesting to understand the affinities between the different donor atoms and the cation, the geometry of the complex and its stoichiometry as well.

The stability of these compounds is confirmed by a whole set of measures: UV-visible titrations and incubations in PBS-buffered media with a minimum percentage of DMSO. We relate this high stability with the results of the in vitro test with human cells and with bacteria, which displayed no activity at all, with few exceptions. Thus, the stability of the compounds could help in predicting that therapeutic use of these complexes is potentially nonsensical unless it is known that the whole molecule can directly interact with a biological target and not behave as a prodrug. On the other hand, they possess encouraging characteristics for radio imaging applications given the stability of their coordinative bonds, other than the advantage of a relatively quick and easy synthesis. Thus, thiosemicarbazones are promising ligands to widen the coordinating chemistry of these cations.

## Figures and Tables

**Table 1:** Summary of the synthetic procedures employed to obtain the coordination compounds. * solvent (methanol or ethanol) depends on the reaction.

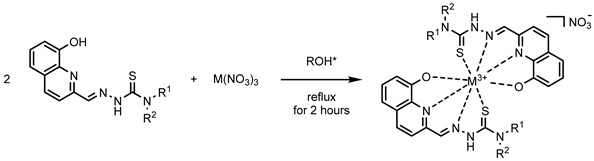		
	M=Ga (Solvent: ROH *)	M=In (Solvent: ROH *)
R^1^=H; R^2^=H	**Ga1** (MeOH)	**In1** (MeOH)
R^1^=Me; R^2^=H	**Ga2** (MeOH)	**In2** (EtOH)
R^1^=Me; R^2^=Me	**Ga3** (MeOH)	**In3** (EtOH)
R^1^=Ph; R^2^=H	**Ga4** (MeOH)	**In4** (EtOH)

**Table 2:** Coordination distances between In^3+^ and donor atoms.

Molecule 1		Molecule 2	
In1-S3	2.6622(4)	In1-S1	2.6223(4)
In1-N11	2.563(1)	In1-N2	2.537(1)
In1-N17	2.329(1)	In1-N13	2.321(1)
In1-O1	2.223(1)	In1-O5	2.253(1)

**Table 3:** Antibiotic assay of ligands and complexes on *E. coli* and *MRSA*.

Compound	MIC *E. coli* [μM]	MIC *MRSA* [μM]
**L1**	>100	>100
**L2**	>100	>100
**L3**	>100	100
**L4**	>100	25
**Ga1**	>100	>100
**In1**	>100	>100
**Ga2**	>100	>100
**In2**	>100	100–50
**Ga3**	>100	50
**In3**	>100	>100
**Ga4**	>100	100
**In4**	>100	>100

## Data Availability

The data presented in this study are available in article or Supplementary Material.

## Supplementary Materials

The following supporting information can be downloaded at: https://www.mdpi.com/article/10.3390/molecules29020497/s1, Figure S1 Number of publications about thiosemicarbazones with biological and medicinal applications be-tween 1918 and 2023. Figure S2. ORTEP representation of **In1.** Figure S3. Hydrogen bond contacts in the crystal structure. Figure S4. Representation of the angle in between the planes comprising the two ligand moieties coordinated to the central indium cation. Table S1. Cytotoxicity assays of **L1**–**L4** and their complexes with gallium (**Ga1**–**Ga4**) and indium (**In1**–**In4**) on human cells belonging to lung cancer cell line A549.

## References

[1] Chitambar C.R. (2010). Medical Applications and Toxicities of Gallium Compounds. Int. J. Environ. Res. Public Health.

[2] Qi J., Deng J., Qian K., Tian L., Li J., He K., Huang X., Cheng Z., Zheng Y., Wang Y. (2017). Novel 2-Pyridinecarboxaldehyde Thiosemicarbazones Ga(III) Complexes with a High Antiproliferative Activity by Promoting Apoptosis and Inhibiting Cell Cycle. Eur. J. Med. Chem..

[3] Yin H.Y., Gao J.J., Chen X., Ma B., Yang Z.S., Tang J., Wang B.W., Chen T., Wang C., Gao S. (2020). A Gallium(III) Complex That Engages Protein Disulfide Isomerase A3 (PDIA3) as an Anticancer Target. Angew. Chem. Int. Ed..

[4] Lessa J.A., Parrilha G.L., Beraldo H. (2012). Gallium Complexes as New Promising Metallodrug Candidates. Inorganica Chim. Acta.

[5] Wang Y., Han B., Xie Y., Wang H., Wang R., Xia W., Li H., Sun H. (2019). Combination of Gallium(III) with Acetate for Combating Antibiotic Resistant: Pseudomonas Aeruginosa. Chem. Sci..

[6] Scaccaglia M., Rega M., Vescovi M., Pinelli S., Tegoni M., Bacci C., Pelosi G., Bisceglie F. (2022). Gallium(III)-Pyridoxal Thiosemicarbazone Derivatives as Nontoxic Agents against Gram-Negative Bacteria. Metallomics.

[7] Kelson A.B., Carnevali M., Truong-Le V. (2013). Gallium-Based Anti-Infectives: Targeting Microbial Iron-Uptake Mechanisms. Curr. Opin. Pharmacol..

[8] Käkelä M., Luoto P., Viljanen T., Virtanen H., Liljenbäck H., Jalkanen S., Knuuti J., Roivainen A., Li X.G. (2018). Adventures in Radiosynthesis of Clinical Grade [68Ga]Ga-DOTA-Siglec-9. RSC Adv..

[9] Kaneta K., Takahama H., Tateishi E., Irie Y., Moriuchi K., Amano M., Okada A., Amaki M., Kiso K., Kanzaki H. (2023). Clinical Outcomes of Radiologic Relapse in Patients with Cardiac Sarcoidosis Under Immunosuppressive Therapies. Am. J. Cardiol..

[10] Matsumura M., Okada A., Yokoyama H., Sekiguchi M., Shimizu A., Tanaka T., Nangaku M., Takano H. (2023). Usefulness of Gallium-67 Scintigraphy for Evaluating the Histopathological Activity in Interstitial Nephritis. Clin. Exp. Nephrol..

[11] Conen P., Pennetta F., Dendl K., Hertel F., Vogg A., Haberkorn U., Giesel F.L., Mottaghy F.M. (2022). [68 Ga]Ga-FAPI Uptake Correlates with the State of Chronic Kidney Disease. Eur. J. Nucl. Med. Mol. Imaging.

[12] Guo Y., Li W., Li H., Xia W. (2019). Identification and Characterization of a Metalloprotein Involved in Gallium Internalization in Pseudomonas Aeruginosa. ACS Infect. Dis..

[13] Oyen W.J.G., Claessens R.A.M.J., Van Horn J.R., Van der Meer J.W.M., Corstens F.H.M. (1990). Scintigraphic Detection of Bone and Joint Infections with Indium-111-Labeled Nonspecific Polyclonal Human Immunoglobulin G. J. Nucl. Med..

[14] Sarko D., Eisenhut M., Haberkorn U., Mier W. (2012). Bifunctional Chelators in the Design and Application of Radiopharmaceuticals for Oncological Diseases. Curr. Med. Chem..

[15] Gray H.W., Cuthbert I., Richards J.R. (1981). Clinical Imaging with Indium-111 Leukocytes: Uptake in Bowel Infarction. J. Nucl. Med..

[16] Makhlouf A., Hajdu I., Hartimath S.V., Alizadeh E., Wharton K., Wasan K.M., Badea I., Fonge H. (2019). 111 In-Labeled Glycoprotein Nonmetastatic b (GPNMB) Targeted Gemini Surfactant-Based Nanoparticles against Melanoma: In Vitro Characterization and in Vivo Evaluation in Melanoma Mouse Xenograft Model. Mol. Pharm..

[17] Shih W.J. (1975). Indium 113m Perfusion Study and the Nonfunctioning Thyroid Nodule. J. Nucl. Med..

[18] Beraldo H. (2020). Pharmacological Applications of Non-Radioactive Indium(III) Complexes: A Field yet to Be Explored. Coord. Chem. Rev..

[19] Pelosi G. (2010). Thiosemicarbazone Metal Complexes: From Structure to Activity. Open Crystallogr. J..

[20] Haribabu J., Balakrishnan N., Swaminathan S., Dorairaj D.P., Azam M., Subarkhan M.K.M., Chang Y.L., Hsu S.C.N., Štarha P., Karvembu R. (2023). Michael Addition-Driven Synthesis of Cytotoxic Palladium(II) Complexes from Chromone Thiosemicarbazones: Investigation of Anticancer Activity through in Vitro and in Vivo Studies. New J. Chem..

[21] Belicchi-Ferrari M., Bisceglie F., Pelosi G., Tarasconi P. (2008). Heterocyclic Substituted Thiosemicarbazones and Their Cu(II) Complexes: Synthesis, Characterization and Studies of Substituent Effects on Coordination and DNA Binding. Polyhedron.

[22] Balakrishnan N., Haribabu J., Dhanabalan A.K., Swaminathan S., Sun S., Dibwe D.F., Bhuvanesh N., Awale S., Karvembu R. (2020). Thiosemicarbazone(s)-Anchored Water Soluble Mono- A Nd Bimetallic Cu(II) Complexes: Enzyme-like Activities, Biomolecular Interactions, Anticancer Property and Real-Time Live Cytotoxicity. Dalt. Trans..

[23] Haribabu J., Srividya S., Mahendiran D., Gayathri D., Venkatramu V., Bhuvanesh N., Karvembu R. (2020). Synthesis of Palladium(II) Complexes via Michael Addition: Antiproliferative Effects through ROS-Mediated Mitochondrial Apoptosis and Docking with SARS-CoV-2. Inorg. Chem..

[24] Lessa J.A., Reis D.C., Mendes I.C., Speziali N.L., Rocha L.F., Pereira V.R.A., Melo C.M.L., Beraldo H. (2011). Antimony(III) Complexes with Pyridine-Derived Thiosemicarbazones: Structural Studies and Investigation on the Antitrypanosomal Activity. Polyhedron.

[25] Gou Y., Wang J., Chen S., Zhang Z., Zhang Y., Zhang W., Yang F. (2016). A−N−heterocyclic Thiosemicarbazone Fe(III) Complex: Characterization of Its Antitumor Activity and Identification of Anticancer Mechanism. Eur. J. Med. Chem..

[26] Reis D.C., Pinto M.C.X., Souza-Fagundes E.M., Wardell S.M.S.V., Wardell J.L., Beraldo H. (2010). Antimony(III) Complexes with 2-Benzoylpyridine-Derived Thiosemicarbazones: Cytotoxicity against Human Leukemia Cell Lines. Eur. J. Med. Chem..

[27] Scaccaglia M., Rega M., Bacci C., Giovanardi D., Pinelli S., Pelosi G., Bisceglie F. (2022). Bismuth Complex of Quinoline Thiosemicarbazone Restores Carbapenem Sensitivity in NDM-1-Positive Klebsiella Pneumoniae. J. Inorg. Biochem..

[28] Hickey J.L., Crouch P.J., Mey S., Caragounis A., White J.M., White A.R., Donnelly P.S. (2011). Copper(II) Complexes of Hybrid Hydroxyquinoline-Thiosemicarbazone Ligands: GSK3β Inhibition Due to Intracellular Delivery of Copper. Dalt. Trans..

[29] Tai Y.X., Ji Y.M., Lu Y.L., Li M.X., Wu Y.Y., Han Q.X. (2016). Cadmium(II) and Indium(III) Complexes Derived from 2-Benzoylpyridine N(4)-Cyclohexylthiosemicarbazone: Synthesis, Crystal Structures, Spectroscopic Characterization and Cytotoxicity. Synth. Met..

[30] Zhang Z., Gou Y., Wang J., Yang K., Qi J., Zhou Z., Liang S., Liang H., Yang F. (2016). Four Copper(II) Compounds Synthesized by Anion Regulation: Structure, Anticancer Function and Anticancer Mechanism. Eur. J. Med. Chem..

