# Supplementary material for: New Stable Gallium(III) and Indium(III) Complexes with Thiosemicarbazone Ligands: A Biological Evaluation
Source: Molecules. 2024 Jan 19;29(2):497. doi: 10.3390/molecules29020497 (PMC10820829; doi:10.3390/molecules29020497)
Supplement: Supplementary file 1 [file molecules-29-00497-s001.zip › molecules-2801755-supplementary.pdf]

molecules

MDPI

*Article*

# New Stable Gallium(III) and Indium(III) Complexes with Thiosemicarbazone Ligands: A Biological Evaluation

**Lorenzo Verderi [1], Mirco Scaccaglia [1], Martina Rega [2], Cristina Bacci [2], Silvana Pinelli [3], Giorgio Pelosi [1,4] and Franco Bisceglie [1,4,*]**

1 Department of Chemistry, Life Sciences and Environmental Sustainability, University of Parma, 43124 Parma, Italy; lorenzo.verderi@unipr.it (L.V.); mirco.scaccaglia@unipr.it (M.S.); giorgio.pelosi@unipr.it (G.P.)
2 Department of Veterinary Science, University of Parma, Strada del Taglio 10, 43126 Parma, Italy; martina.rega@unipr.it (M.R.); cristina.bacci@unipr.it (C.B.)
3 Department of Medicine and Surgery, University of Parma, Via Gramsci 14, 43126 Parma, Italy; silvana.pinelli@unipr.it
4 Centre of Excellence for Toxicological Research (CERT), University of Parma, 43124 Parma, Italy
* Correspondence: franco.bisceglie@unipr.it

## Supplementary Materials

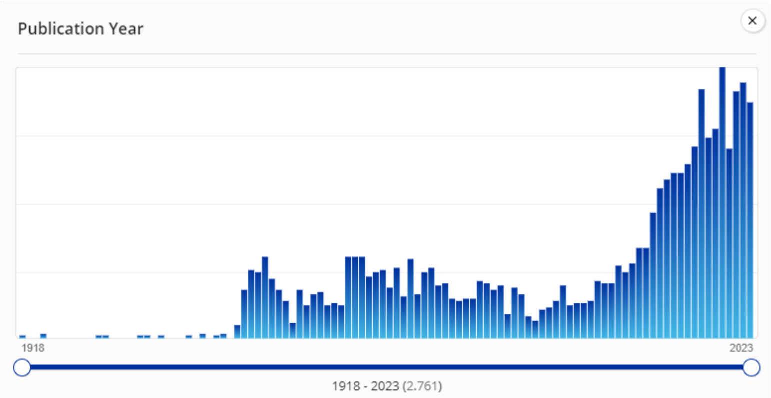


**Figure S1** Number of publications about thiosemicarbazones with biological and medicinal applications between 1918 and 2023, from *Scifinder-n.org*.

## Crystal Data for In1

C28 H38 In N9 O9 S2, monoclinic, *P*-1, a= 11.1212(7)Å, b= 12.2192(8)Å, c=14.1962(9)Å, $\alpha$ = 106.770(2)°, $\beta$ = 103.047(2)°, $\gamma$ =100.111(2)°; V= 1738.5(2)Å$^3$; Z=2; dcalc=1.573 mg/cm$^3$ , F(000)= 844, MoK$\alpha$ radiation ($\lambda$ = 0.71073), mu=0.864, Tot. refl.= 66284, hkl range= -15<h<15, -16<k<16, -19<l<19; Theta range 2.28-25.24, unique reflections= 9008, number of parameters = 461 , GooF= 1.009, R=0.023, wR2=0.064, CSD 2311577

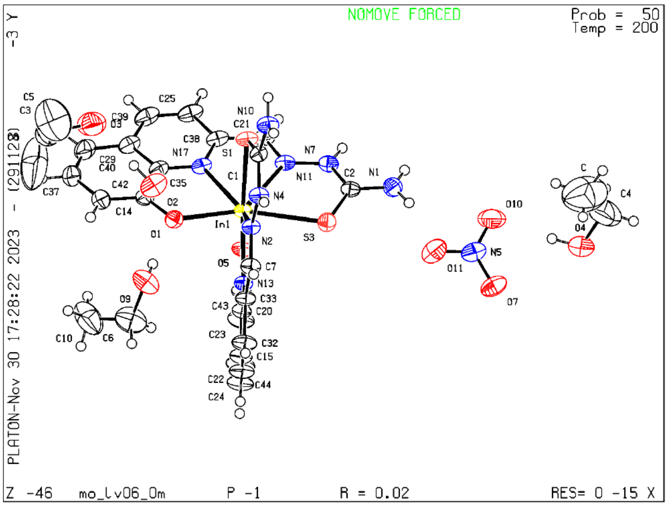


**Figure S2** ORTEP representation of **In1**

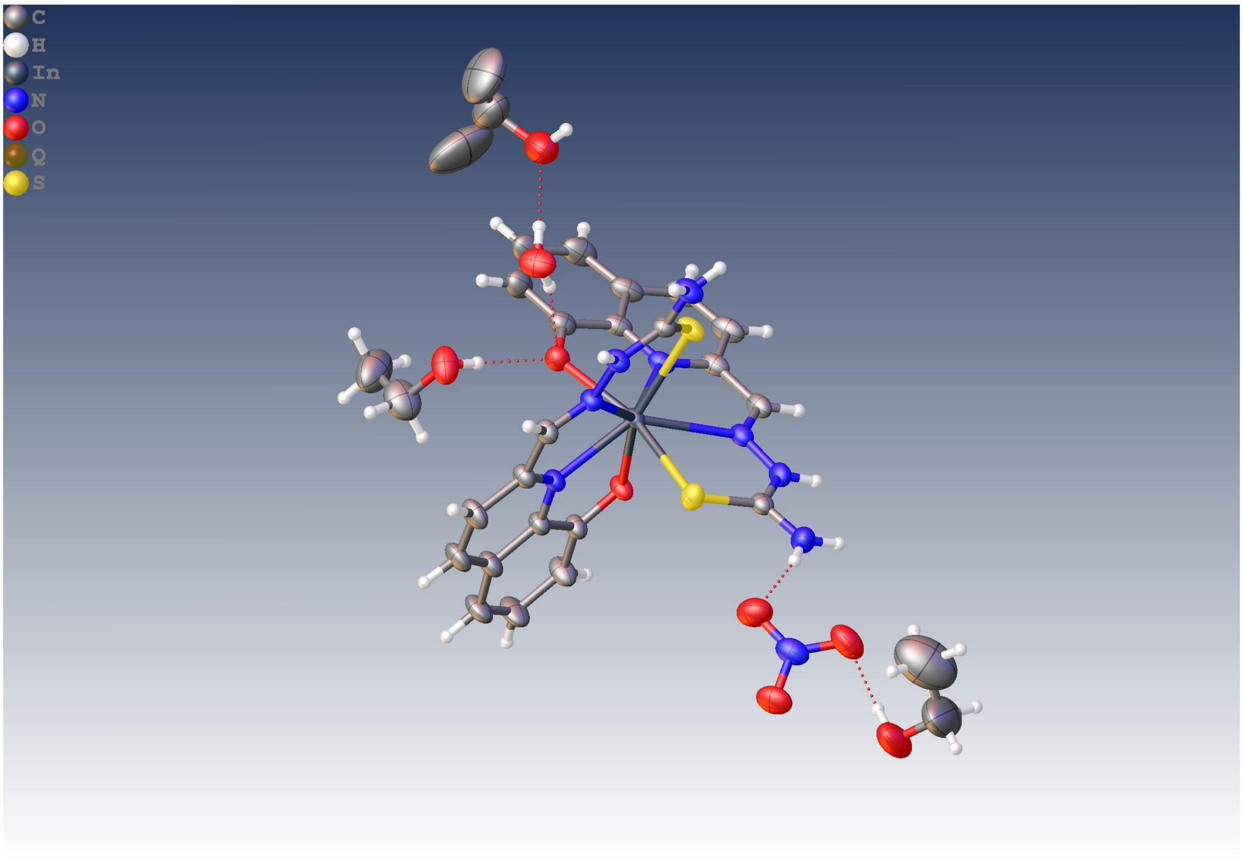


**Figure S3** Hydrogen bond contacts in the crystal structure.

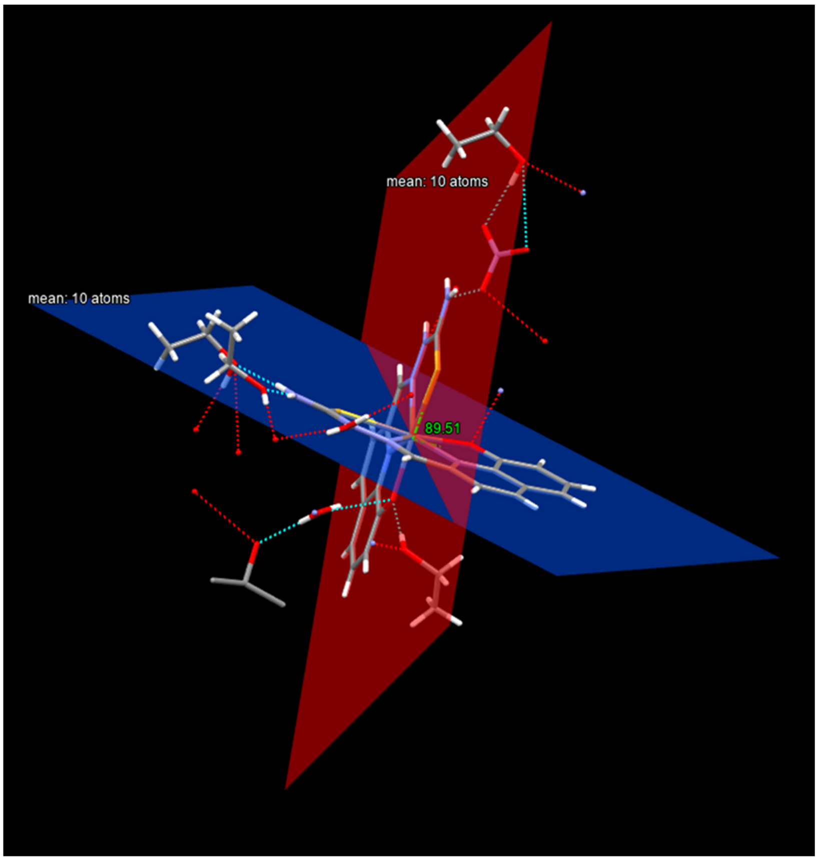


**Figure S4** Representation of the angle in between the planes comprising the two ligand moieties coordinated to the central indium cation.

**Table S1.** Cytotoxicity assays of **L1-L4** and their complexes with gallium (**Ga1-Ga4**) and indium (**In1-In4**) on human cells belonging to lung cancer cell line A549.

| LIGAND | Ga(III) COMPLEX | In(III) COMPLEX |
|---|---|---|
| 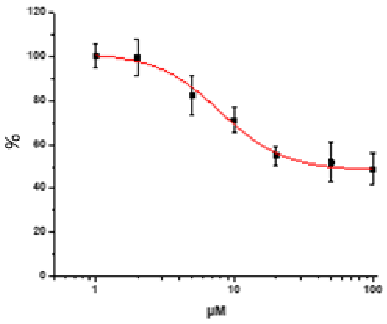 | 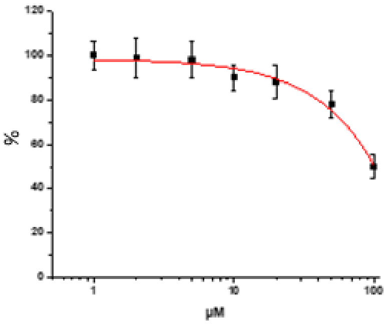 | 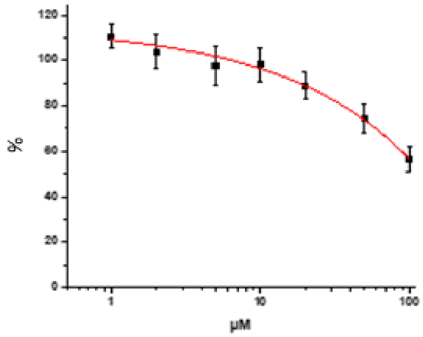 |
| **L1** $IC_{50}$: 50 µM ± 8 | **Ga1** $IC_{50}$: 100 µM ± 6 | **In1** $IC_{50}$: >100 µM |

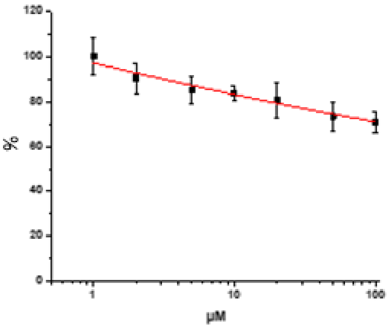


**L2** $IC_{50}$: >100 µM

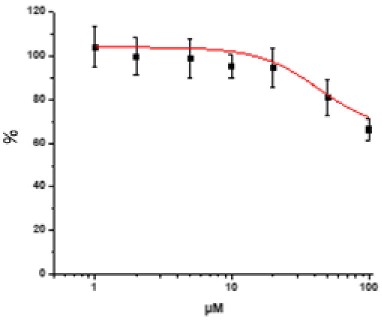


**Ga2** $IC_{50}$: >100 µM

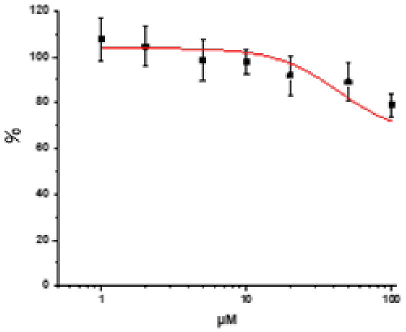


**In2** $IC_{50}$: >100 µM

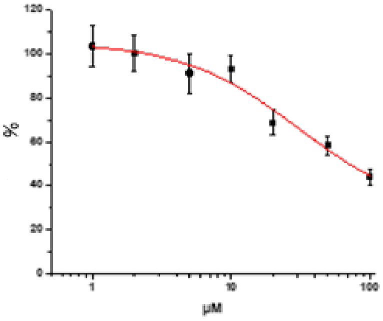


**L3** $IC_{50}$: 71 µM ± 1

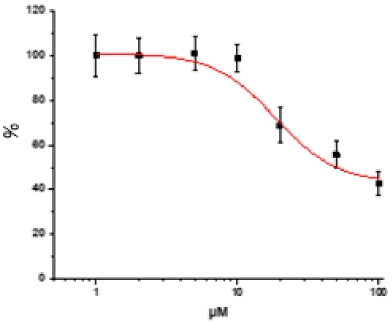


**Ga3** $IC_{50}$: 50 µM ± 1

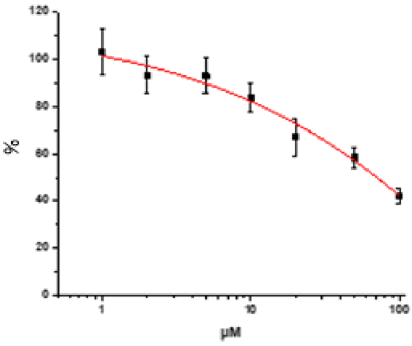


**In3** $IC_{50}$: 65 µM ± 2

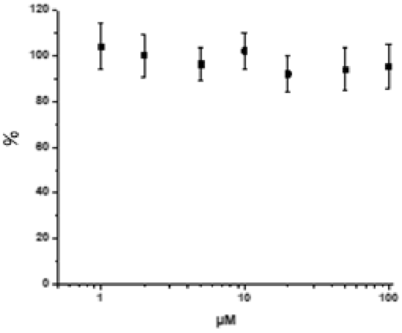


**L4** $IC_{50}$: >100 µM

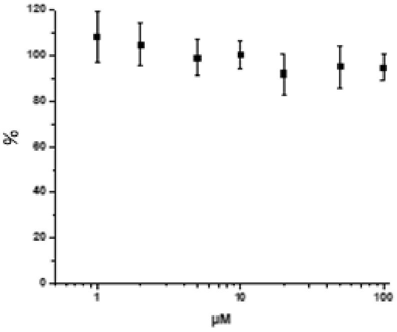


**Ga4** $IC_{50}$: >100 µM

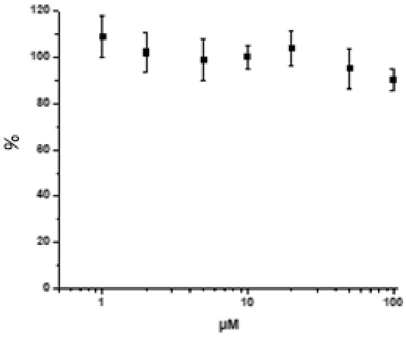


**In4** $IC_{50}$: >100 µM

**Ligands Preparation**

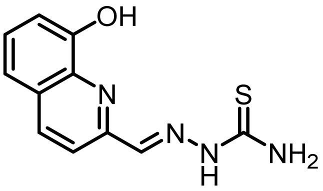


**L1**

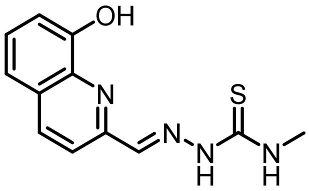


**L2**

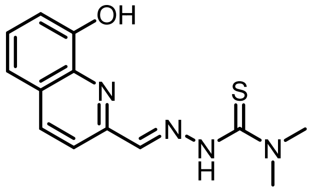

**L3**

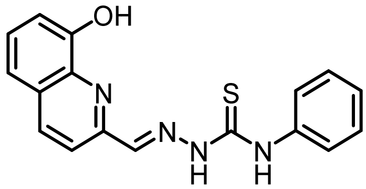

**L4**

The ligands were synthesized following the procedure of our previous article[23], in which the full characterization data of the compounds are available.

(((8-Hydroxyquinolin-2-yl)methylidene)amino)thiourea (**L1**). 8-Hydroxy-2-quinolinecarboxaldehyde (192 mg, 1.11 mmol) and acetic acid (cat.) were dissolved in refluxing methanol (10 mL). As the mixture became clear, a solution of thiosemicarbazide (107 mg, 1.17 mmol) 6 mL was added to it. Precipitation then occurred, and the solution was left cooling to 0°C. The product was separated via filtration, yielding a white powder (44%). $^1$H-NMR (400 MHz, DMSO-$d_6$): [ppm] 11.92 (s, 1H, C=N-N*H*); 9.84 (s, 1H, O*H*); 8.80 (m, 1H, N*H*(CH3)); 8.42 (d, 1H, C*H* arom.); 8.31 (d, 1H, C*H* arom.); 8.27 (s, 1H, C*H*=N); 7.44 (t, 1H, C*H* arom.); 7.39 (dd, 1H, C*H* arom.); 7.11 ppm (dd, 1H, C*H* arom.); 3.07 (d, 3H, NH(C$H_3$)).

1-(((8-Hydroxyquinolin-2-yl)methylidene)amino)-3-methylthiourea (**L2**). 8-Hydroxy-2-quinolinecarboxaldehyde (166 mg, 0.96 mmol) was dissolved in ethanol (10 mL) stirring at RT. As the mixture became clear, a solution of 4-Methylthiosemicarbazide (101 mg, 0.96 mmol) and acetic acid (cat.) in 10 mL was added to it. The mixture was then left stirring at reflux overnight. Precipitation then occurred, and the solution was left cooling to 0°C. The product was separated via filtration, yielding a white powder (83%). $^1$H-NMR (400 MHz, DMSO-$d_6$): [ppm] 11.92 (s, 1H, C=N-N*H*); 9.84 (s, 1H, O*H*); 8.80 (m, 1H, N*H*(CH3)); 8.42 (d, 1H, C*H* arom.); 8.31 (d, 1H, C*H* arom.); 8.27 (s, 1H, C*H*=N); 7.44 (t, 1H, C*H* arom.); 7.39 (dd, 1H, C*H* arom.); 7.11 ppm (dd, 1H, C*H* arom.); 3.07 (d, 3H, NH(C$H_3$)).

1-(((8-Hydroxyquinolin-2-yl)methylidene)amino)-3,3-dimethylthiourea (**L3**). 8-Hydroxy-2-quinolinecarboxaldehyde (131 mg, 0.76 mmol) and acetic acid (cat.) were dissolved in ethanol (10 mL) stirring at RT. As the mixture became clear, a solution of 4,4-Dimethylthiosemicarbazide (89 mg, 0.75 mmol) in 10 mL was added to it. The mixture was then left stirring at RT overnight. Precipitation then occurred, and the solution was left cooling to 0°C. The product was separated via filtration, yielding a yellow-white powder (83%). $^1$H-NMR (400 MHz, DMSO-$d_6$): [ppm] 11.40 (s, 1H, C=N-N*H*); 9.78 (s, 1H, O*H*); 8.39 (s, 1H, C*H*=N); 8.30 (d, 1H, C*H* arom.); 8.03 (d, 1H, C*H* arom.); 7.44 (t, 1H, C*H* arom.); 7.39 (d, 1H, C*H* arom.); 7.12 (dd, 1H, C*H* arom.).

3-(((8-Hydroxyquinolin-2-yl)methylidene)amino)-1-phenylthiourea (**L4**). 8-Hydroxy-2-quinolinecarboxaldehyde (106 mg, 0.61 mmol) and acetic acid (cat.) were dissolved in ethanol (6 mL) stirring at RT. As the mixture became clear, a solution of 4-Phenylthiosemicarbazide (104 mg, 0.62 mmol) in 10 mL was added to it. The mixture was then left stirring at RT overnight. Precipitation then occurred, and the solution was left cooling to 0°C. The product was separated via filtration, yielding a yellow powder (86%). $^1$H NMR (400 MHz, DMSO-$d_6$): [ppm] 12.25 (s, 1H, C=N-N*H*); 10.38 (s, 1H, S=C-N*H*-Ph); 9.91 (s, 1H, O*H*); 8.60 (d, 1H, C*H* arom. (Quinoline)); 8.38 (s, 1H, C*H*=N); 8.31 (d, 1H, C*H* arom. (Quinoline)); 7.56 (d, 2H, C*H* arom. (Quinoline)); 7.44 (m, 4H, C*H* arom. (Quinoline + Phenyl group)); 7.26 (t, 1H, C*H* arom. (Quinoline)); 7.12 (d, 1H, C*H* arom. (Phenyl group)).

## Complexes Characterization Spectra

**Ga1**

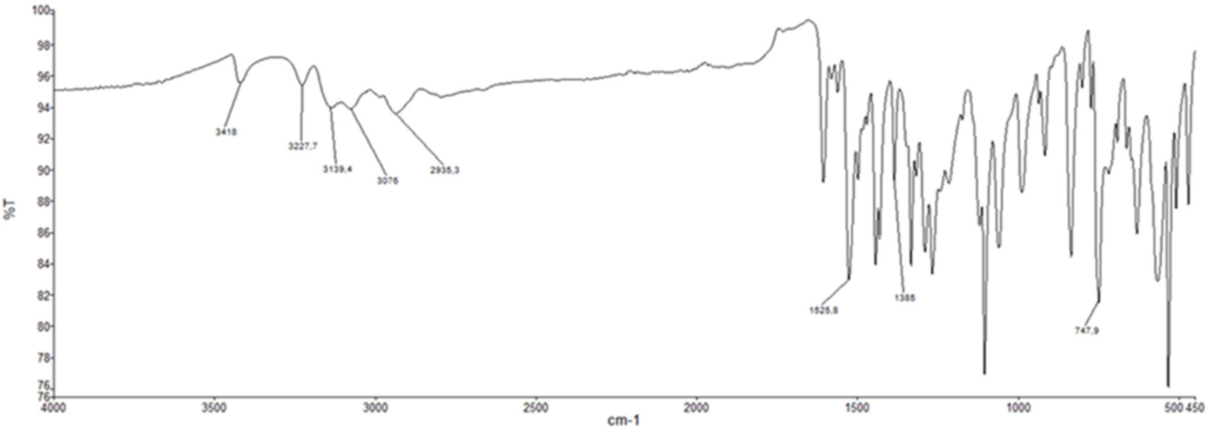


IR (ATR, cm$^{-1}$): 3418, 3228, 3139, 3076 and 2935 w (N-H, O-H and C-H), 1526 s (C=N), 1385 m and 745 s (C=S).

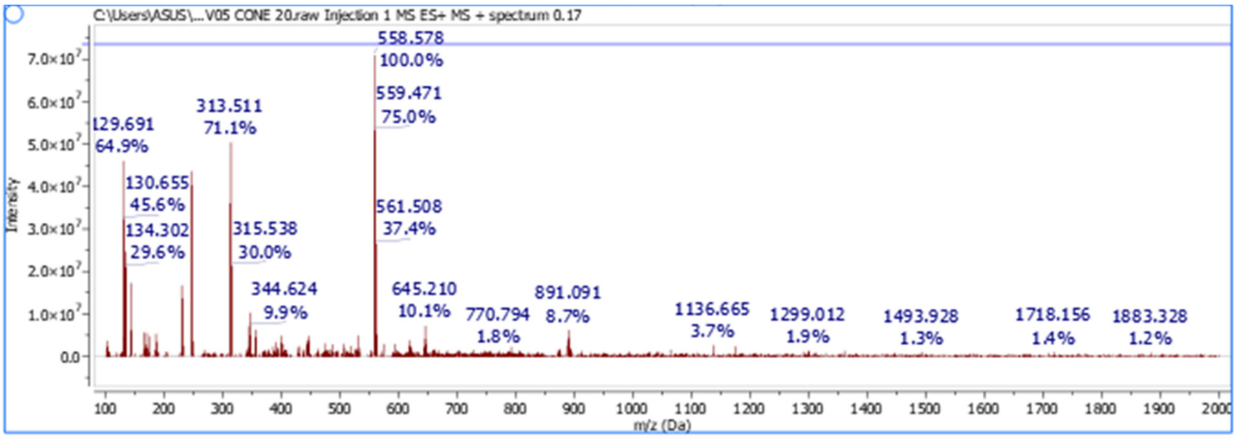


**CONE 20 V** ESI-MS *m/z* (%): 313.5 ([Ga+L-2H]$^{+}$ 71.1), 558.6 ([Ga+2L-2H]$^{+}$ 100.0).

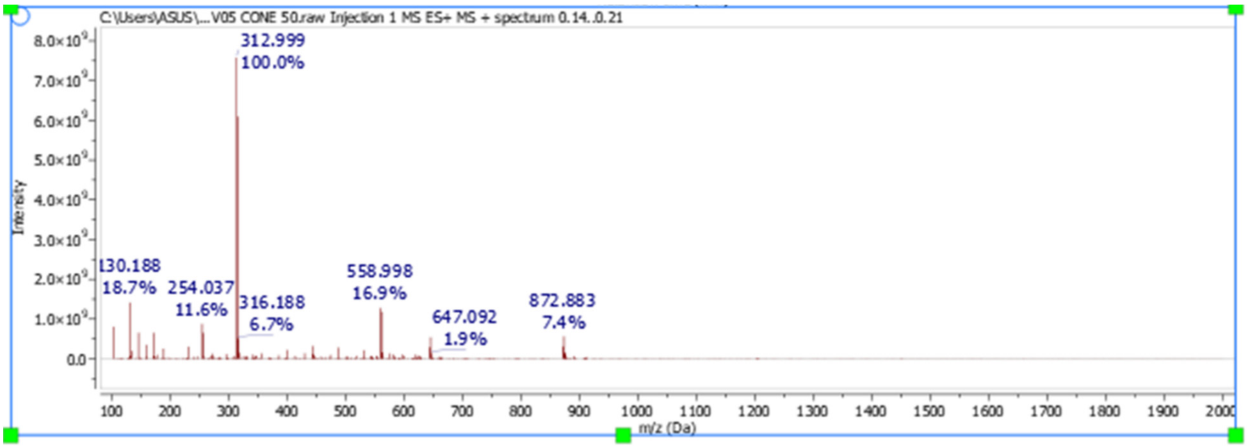


**CONE 50 V** ESI-MS *m*/*z* (%): 313.0 ([Ga+L-2H]$^+$ 100.0), 559.0 ([Ga+2L-2H]$^+$ 16.9).

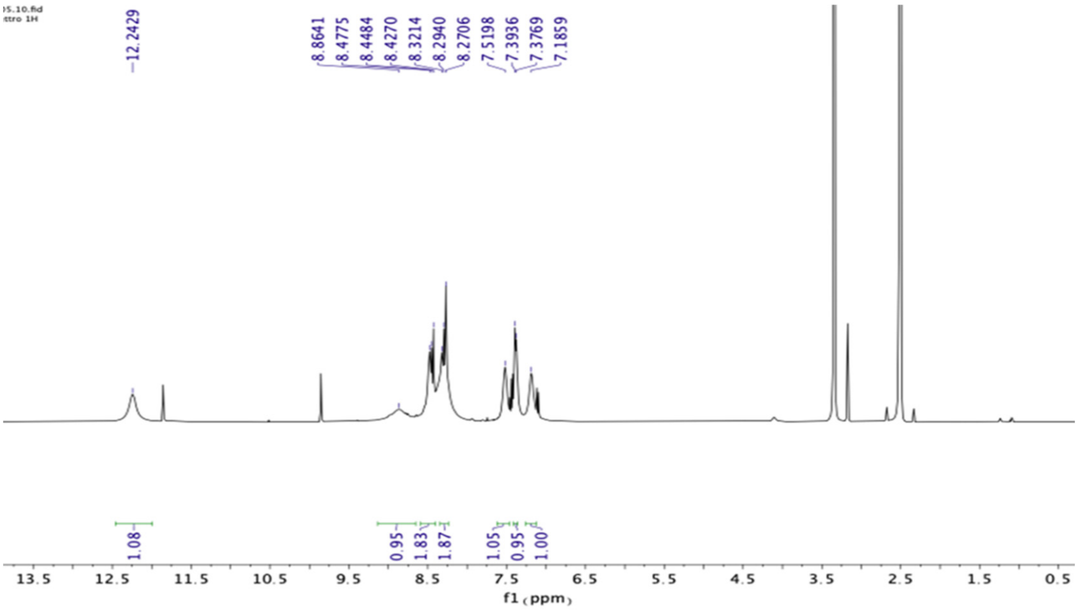


$^{1}$H-NMR (400 MHz, DMSO): [ppm] 12.24 (s, 1H, N**H**), 8.86 (br, 1H, N=C-**H**), 8.48 (br, 1H, C**H** ar.), 8.44 (d, 1H, C**H** ar.), 8.32 (br, 1H, C**H** ar.), 8.29 (t, 1H, C**H** ar.), 7.52 (s, 1H, N**H**), 7.38 (d, 1H, C**H** ar.), 7.19 (s, 1H, N**H**).

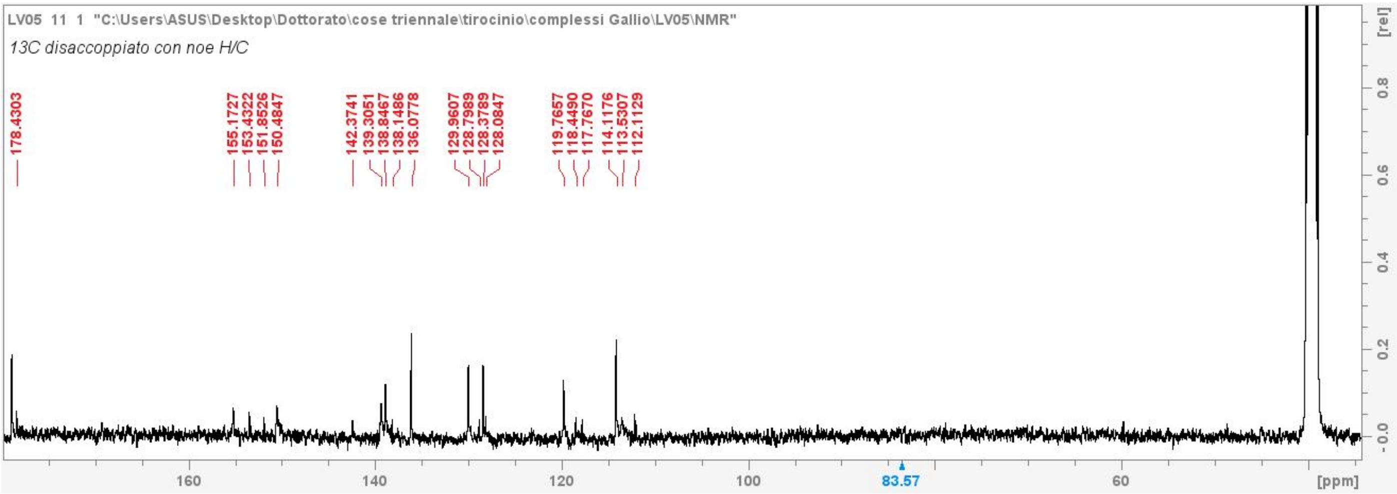

$^{13}$C-NMR (400 MHz, DMSO): [ppm] 178.4 (**C**=N aliph.), 155.2 (**C**=S), 153.4 (**C**-OH), 151.9-150.5 (**C**=N ar.), 142.4 (C=**C**=N ar.), 139.3-138.8-138.1 (C=**C**=C ar.), 136.1 (C=**C**H-C ar., N-para), 130.0-128.8 (C=**C**H-C ar., OH-para), 128.4-128.1 (C=**C**H-C ar., N-meta), 120.0-118.4-117.8 (C=**C**H-C ar., OH-para), 114.1-113.5-112.1 (C=**C**H-C ar., OH-ortho).

**In1**

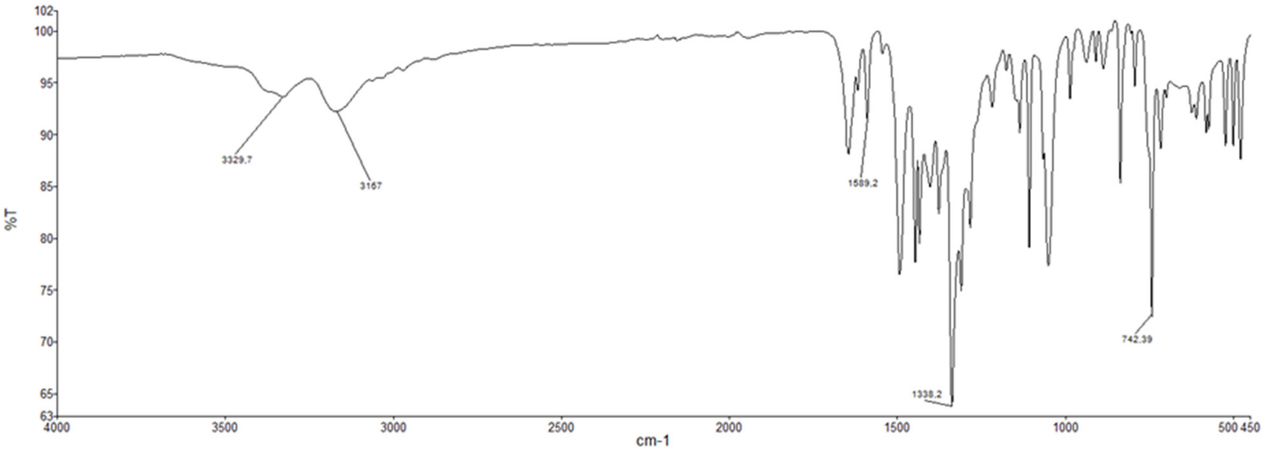


IR (ATR, cm$^{-1}$): 3330 and 3167 w (N-H, O-H and C-H), 1589 m (C=N), 1138 and 742 s (C=S).

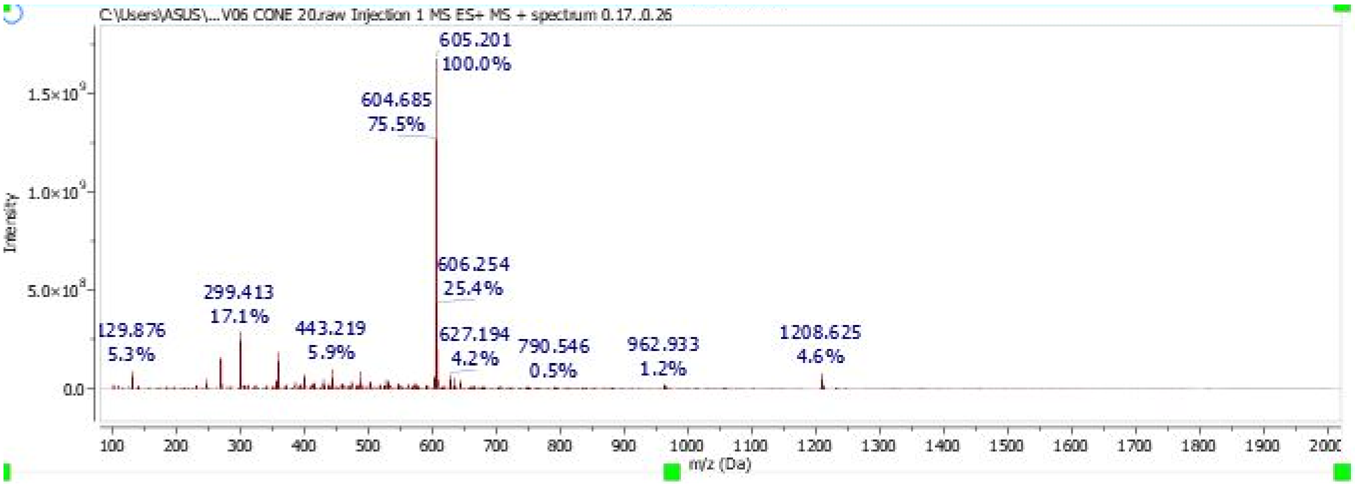


**CONE 20 V** ESI-MS *m*/*z* (%): 605.2 ([In+2L-2H]$^+$, 100.0).

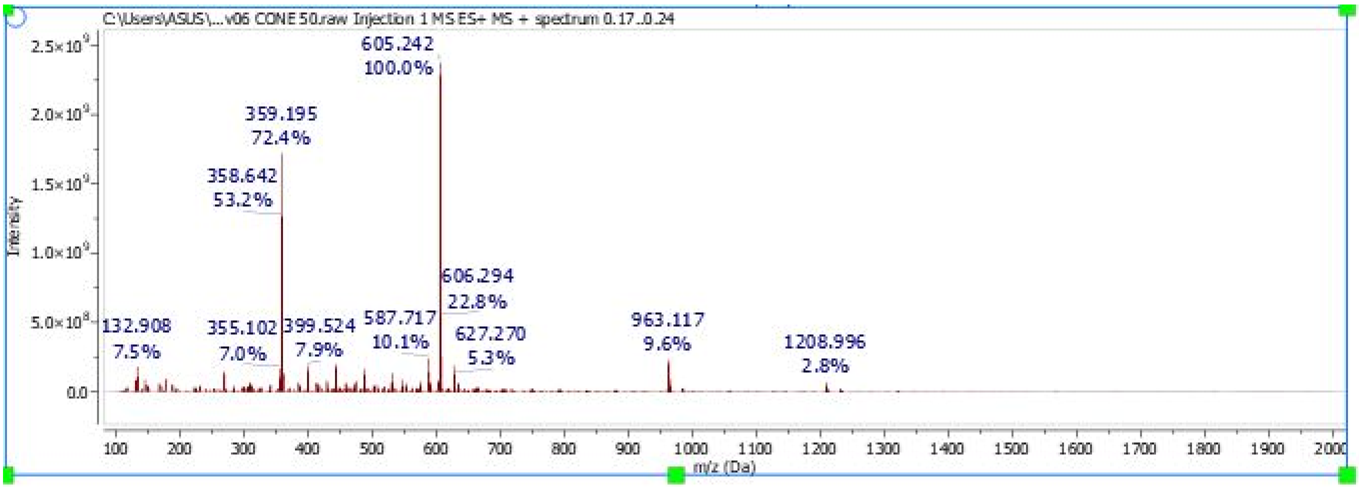


**CONE 50 V** ESI-MS *m*/*z* (%): 359.2 ([In+L-2H]$^+$, 72.4) 605.2 ([In+2L-2H]$^+$, 100.0).

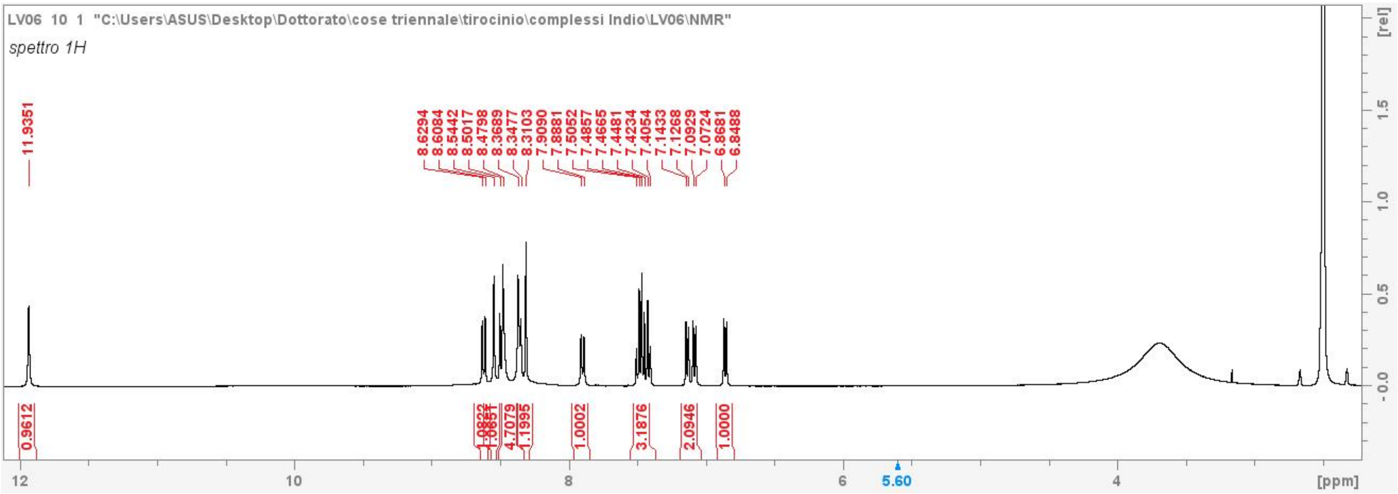


$^1$H-NMR (400 MHz, DMSO): [ppm] 11.94 (s, 1H, HC=N-N**H**), 8.62 (d, 1H, C**H** ar.), 8.54 (s, 1H, N**H**), 8.52-8.33 (m, 5H, C**H** ar.+N**H**), 8.31 (s, 1H, N=C-**H**), 7.90 (d, 1H, C**H** ar.), 7.53-7.38 (m, 3H, C**H** ar.), 7.17-7.04 (m, 2H, C**H** ar.), 6.86 (d, 1H, C**H** ar.).

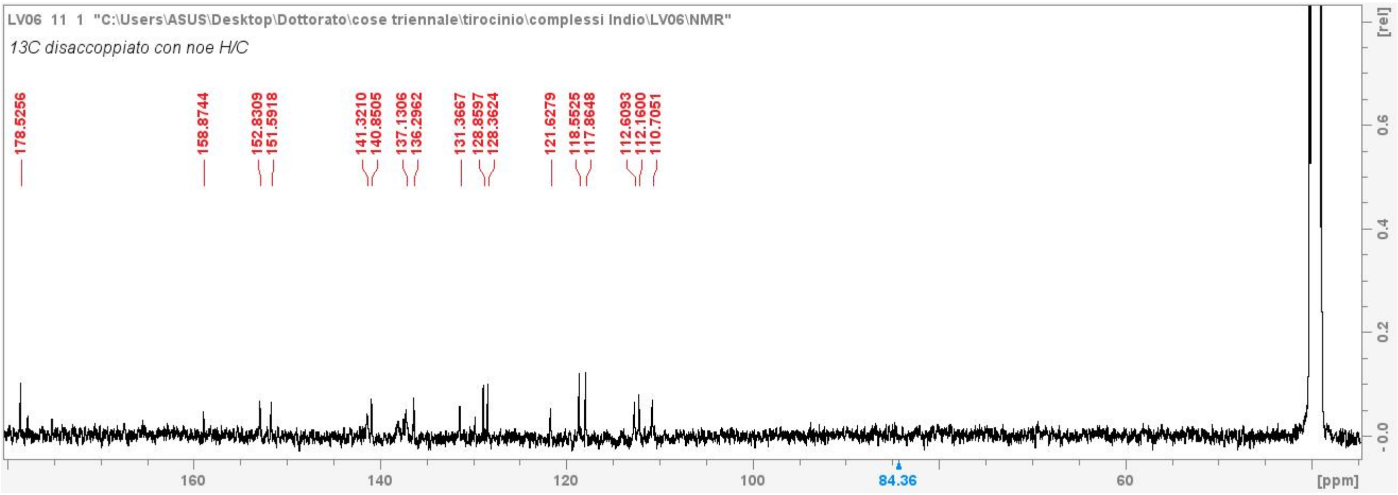

$^{13}$C-NMR (400 MHz, DMSO): [ppm] 178.5 (**C**=N aliph.), 158.9 (**C**=S), 152.8 (**C**-OH), 151.6 (**C**=N ar.), 141.3-140.8 (C=**C**=N ar.), 137.1 (C=**C**=C ar.), 136.3 (C=**C**H-C ar., N-para), 131.4 (C=**C**H-C ar., OH-para), 128.9-128.4 (C=**C**H-C ar., N-meta), 121.6-118.6-117.9 (C=**C**H-C ar., OH-para), 112.6-112.2-110.7 (C=**C**H-C ar., OH-ortho).

**Ga2**

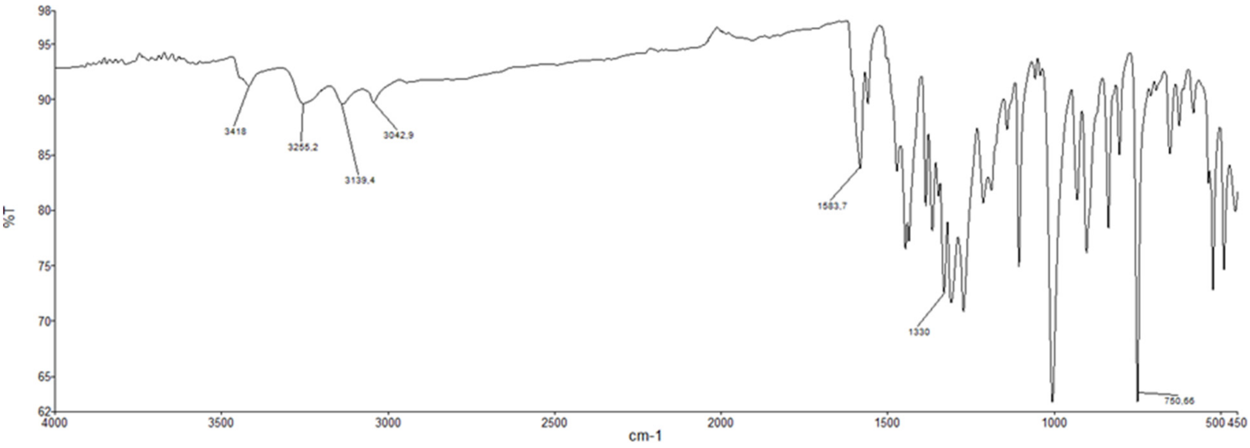


IR (ATR, cm[-1]): 3418, 3255, 3139, 3042 w (N-H, O-H and C-H), 1584 (C=N), 1130 and 751 s (C=S).

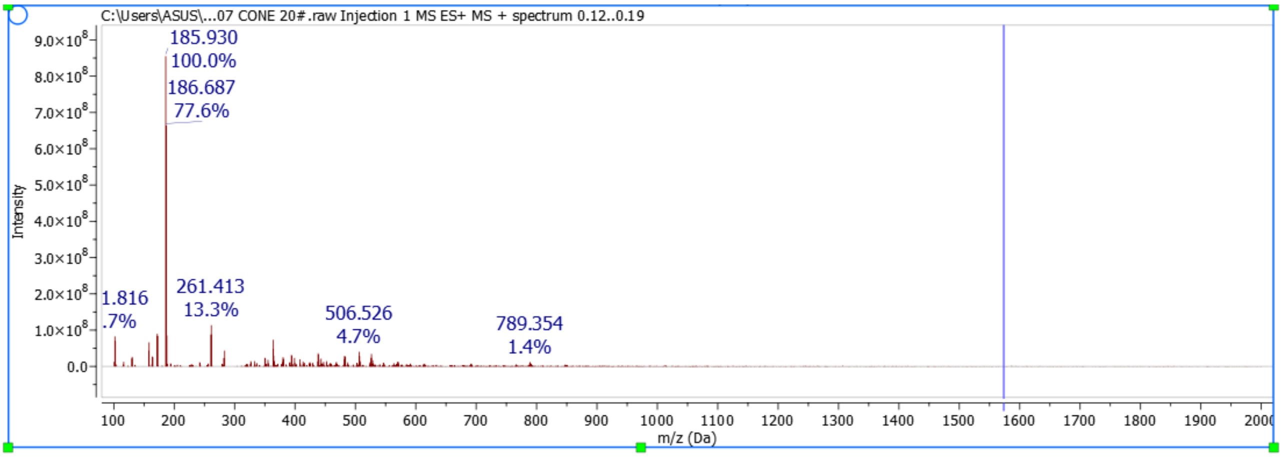


**CONE 20 V** ESI-MS *m*/*z* (%): 326.8 ([Ga+L-2H]$^+$, 1.7).

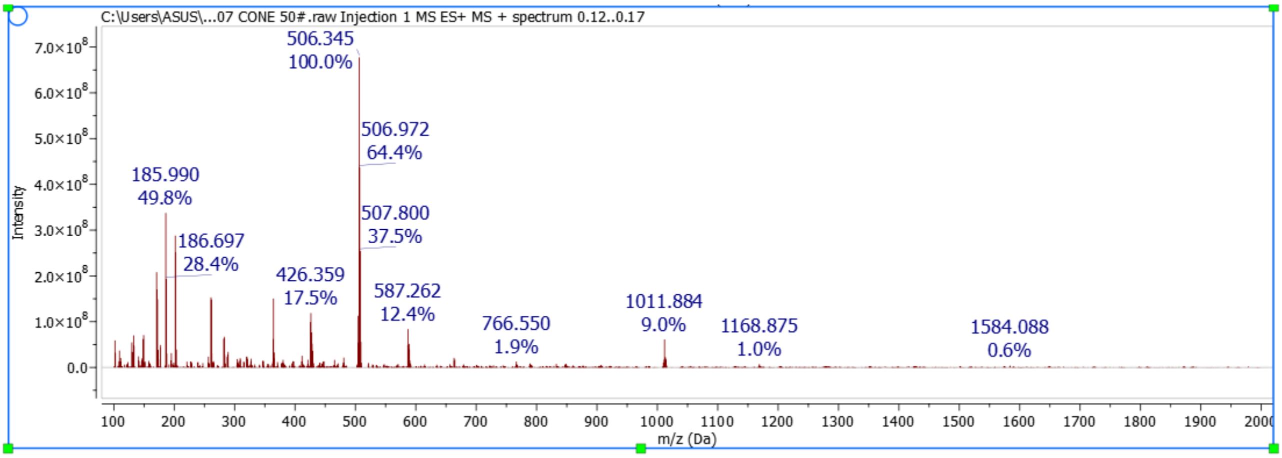


**CONE 50 V** ESI-MS *m*/*z* (%): 587.3 ([Ga+2L-2H]$^{+}$, 12.4).

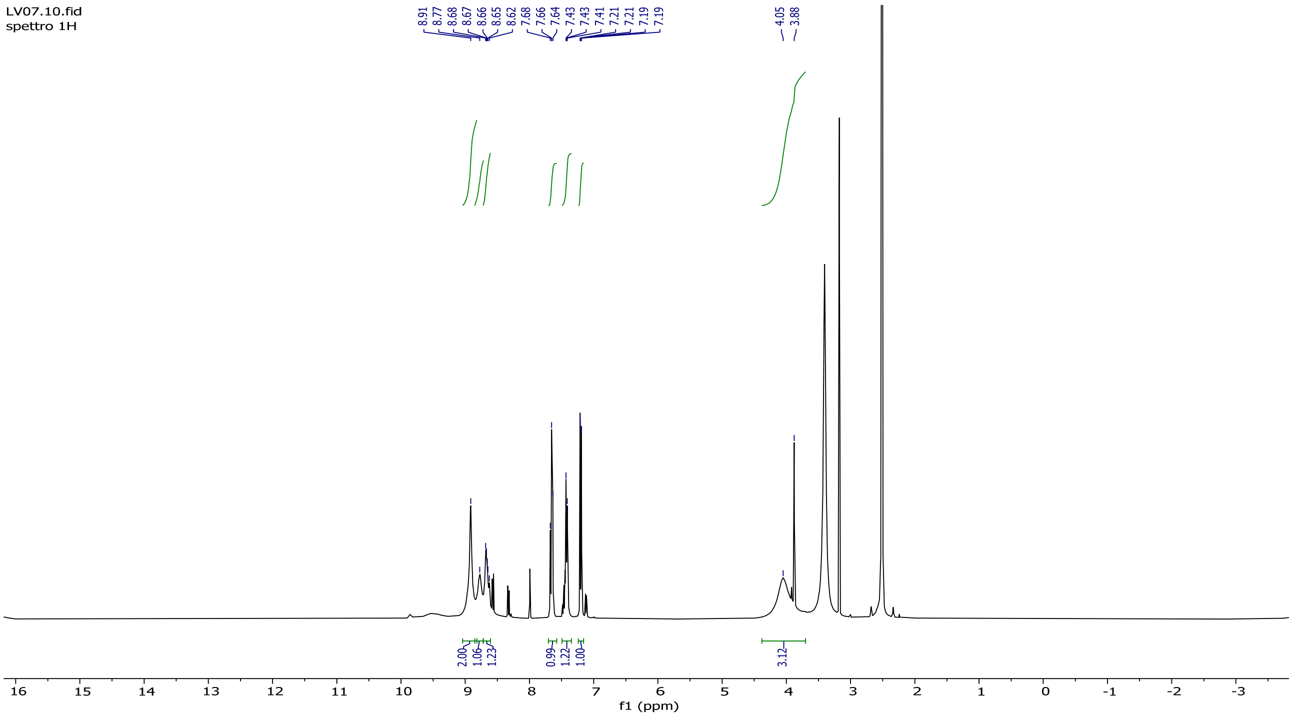


$^{1}$H-NMR (400 MHz, DMSO): [ppm] 8.91 (s, 1H, N=C-**H**), 8.91 (br, 1H, N**H**), 8.77 (s, 1H, HC=N-N**H**), 8.66 (br, 1H, C**H** ar.), 7.66 (t, 1H, **CH** ar.), 7.42 (dd, 1H, **CH** ar.), 7.20 (dd, 1H, **CH** ar.), 4.05 (s, 3H, **CH**$_3$).

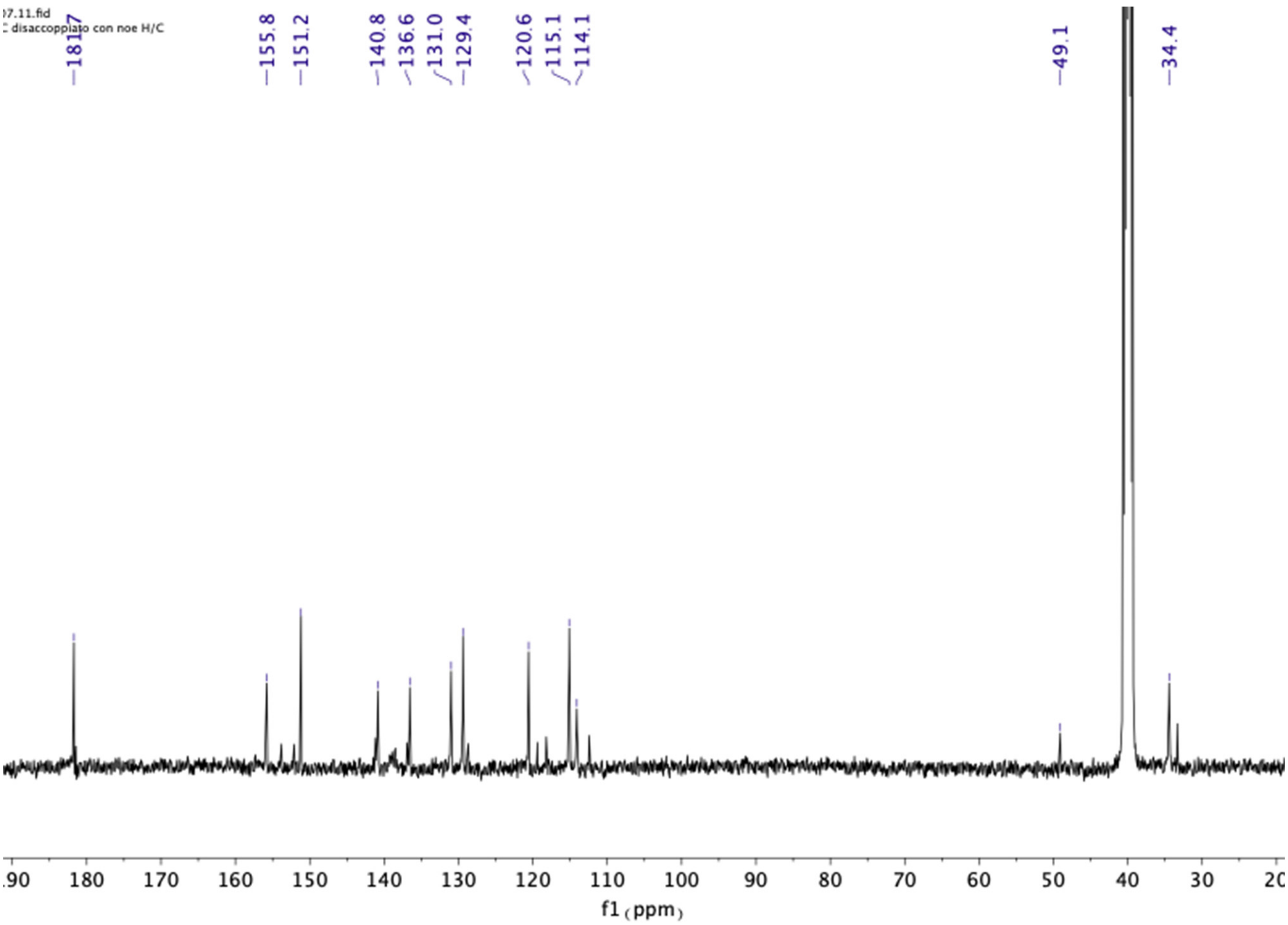


$^{13}$C-NMR (400 MHz, DMSO): [ppm] 181.7 (**C**=N aliph.), 155.8 (**C**=S), 151.2 (**C**-OH), 140.8 (**C**=N ar.), 136.6 (C=**C**=N ar.), 131.0 (C=**C**=C ar.), 129.4 (C=**C**H-C ar., N-para), 120.6 (C=**C**H-C ar., OH-para), 115.1 (C=**C**H-C ar., N-meta), 114.1 (C=**C**H-C ar., OH-para), 49.1 (C=**C**H-C ar., OH-ortho), 34.4 (**C**$H_3$).

**In2**

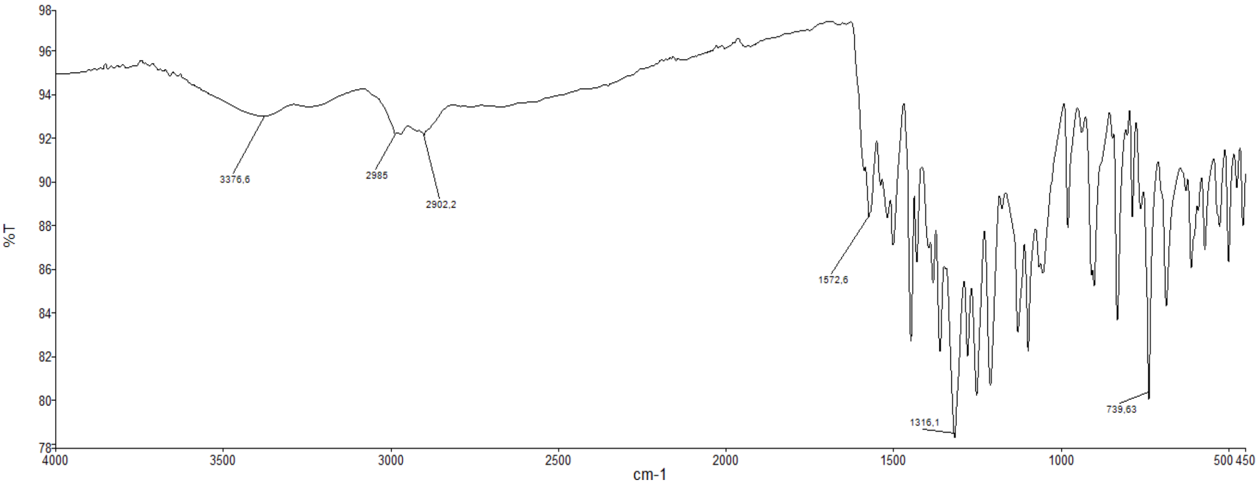


IR (ATR, cm$^{-1}$): 3377, 2985 and 2902 w (C-H, O-H), 1573 m (C=N), 1316 and 740 s (C=S).

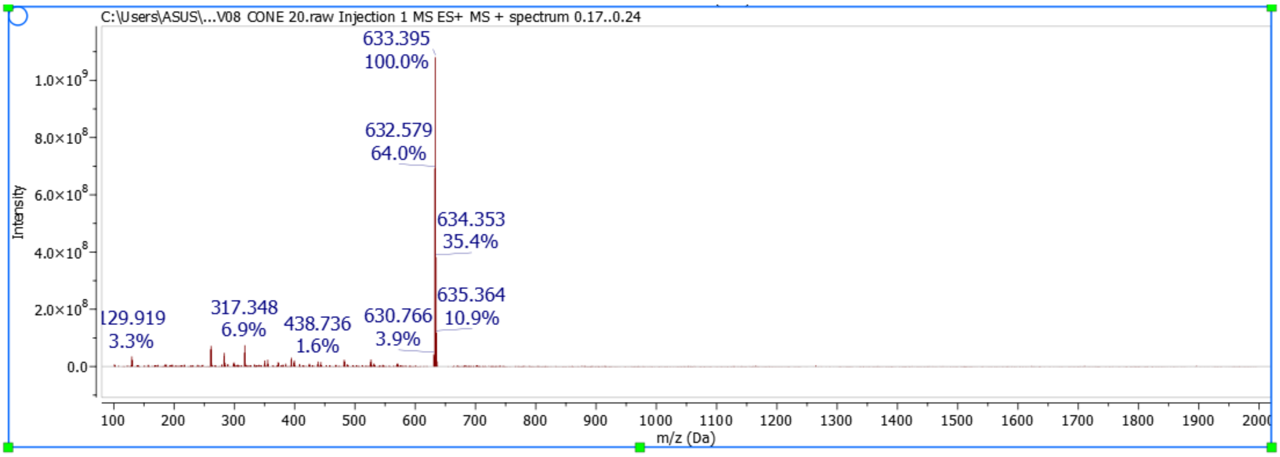


**CONE 20 V** ESI-MS *m/z* (%): 633.4 ([In+2L-2H]$^{+}$, 100.0).

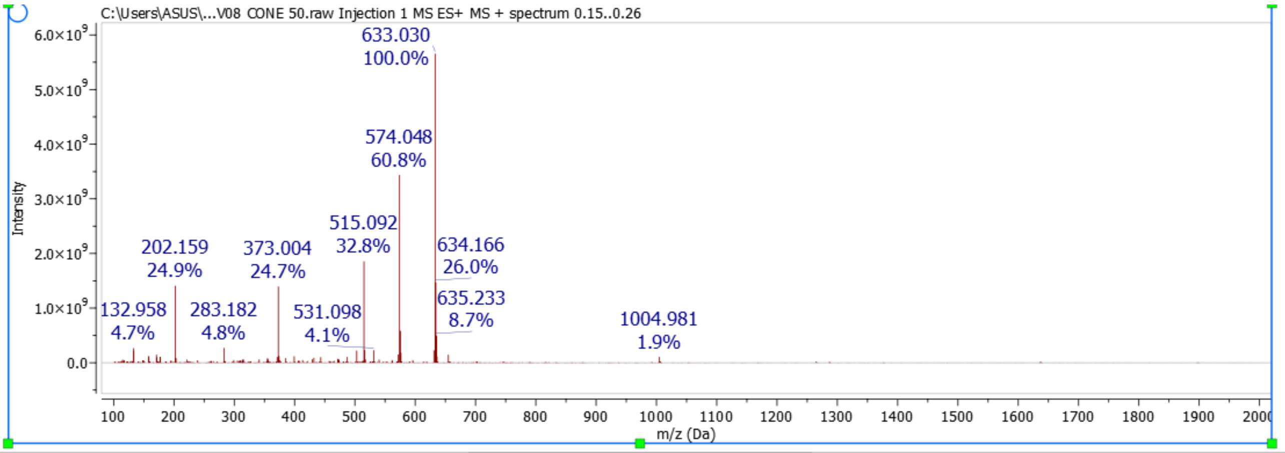


**CONE 50 V** ESI-MS *m/z* (%): 633.0 ([In+2L-2H]$^{+}$, 100.0), 373.0 ([In+L-2H]$^{+}$, 24.7).

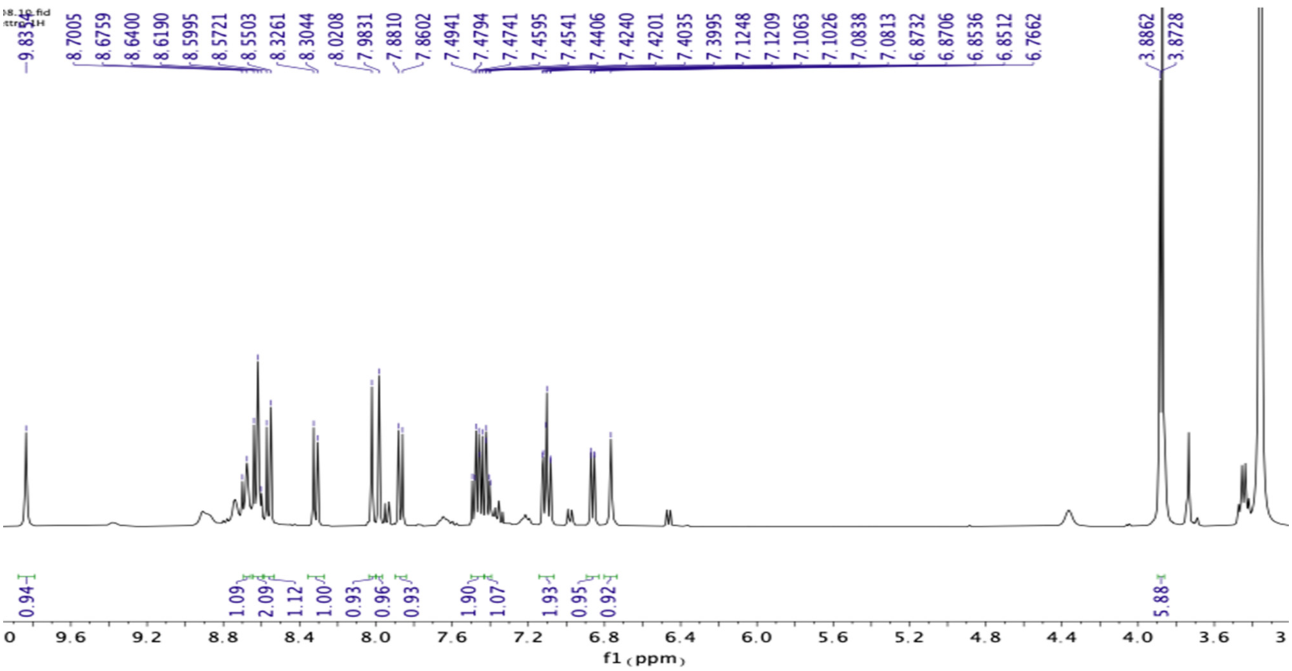


$^1$H-NMR (400 MHz, DMSO): [ppm] 9.84 (s, 1H, HC=N-N**H**), 8.91 (s, 1H, N**H**), 8.68 (s, 1H, N**H**), 8.63 (br, 1H, **CH** ar.), 8.62 (s, 1H, N-**H**), 8.56 (d, 1H, **CH** ar.), 8.32 (d, 1H, **CH** ar.), 8.02 (s, 1H, N=C-**H**), 7.98 (s, 1H, N=C-**H**), 7.87 (d, 1H, **CH** ar.), 7.48 (t, 1H, **CH** ar.), 7.45 (dd, 1H, **CH** ar.), 7.41 (dd, 1H, **CH** ar.), 7.11 (dd, 1H, **CH** ar.), 7.10 (t, 1H, **CH** ar.), 6.86 (dd, 1H, **CH** ar.), 6.77 (s, 1H, N**H**), 3.89 (s, 3H, $CH_3$), 3.88 (s, 3H, $CH_3$).

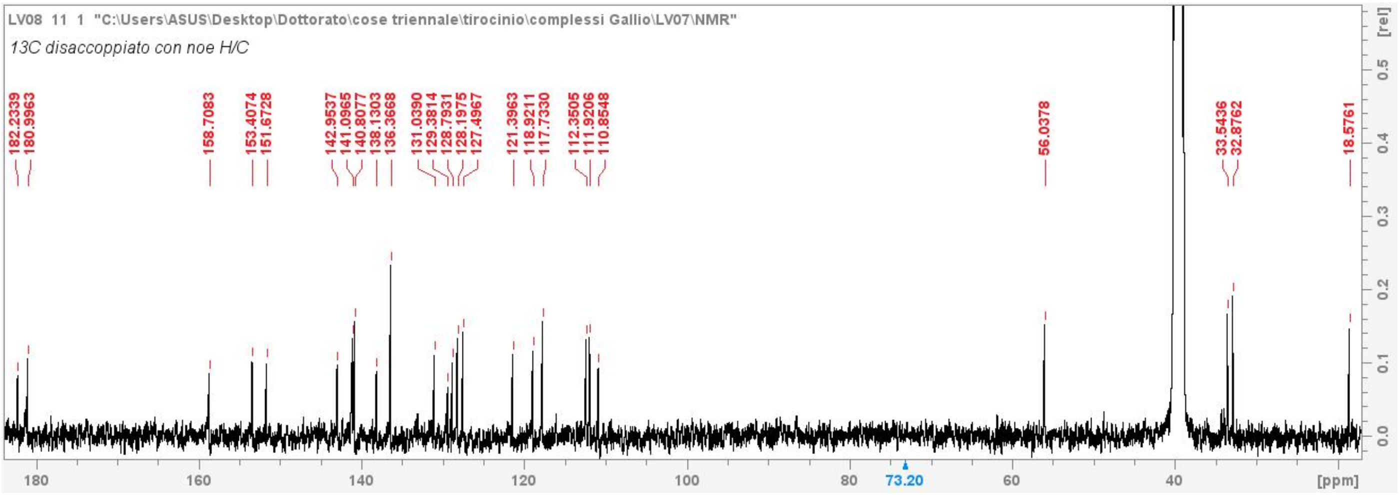


$^{13}$C-NMR (400 MHz, DMSO): [ppm] 182.2, 181.0 (**C**=N aliph.), 158.7 (**C**=S), 153.4 (**C**-OH), 151.7 (**C**=N ar.), 143.0, 141.1, 140.8 (C=**C**=N ar.), 138.1, 136.4 (C=**C**=C ar.), 131.0 , 129.4 (C=**C**H-C ar., N-para), 128.8, 128.2, 127.5 (C=**C**H-C ar., OH-para), 121.4, 118.9 (C=**C**H-C ar., N-meta), 117.7 (C=**C**H-C ar., OH-para), 112.4, 111.9, 110.9 (C=**C**H-C ar., OH-ortho), 33.5, 32.9 (**C**$H_3$).

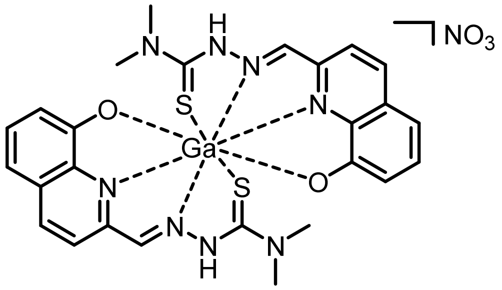


**Ga3**

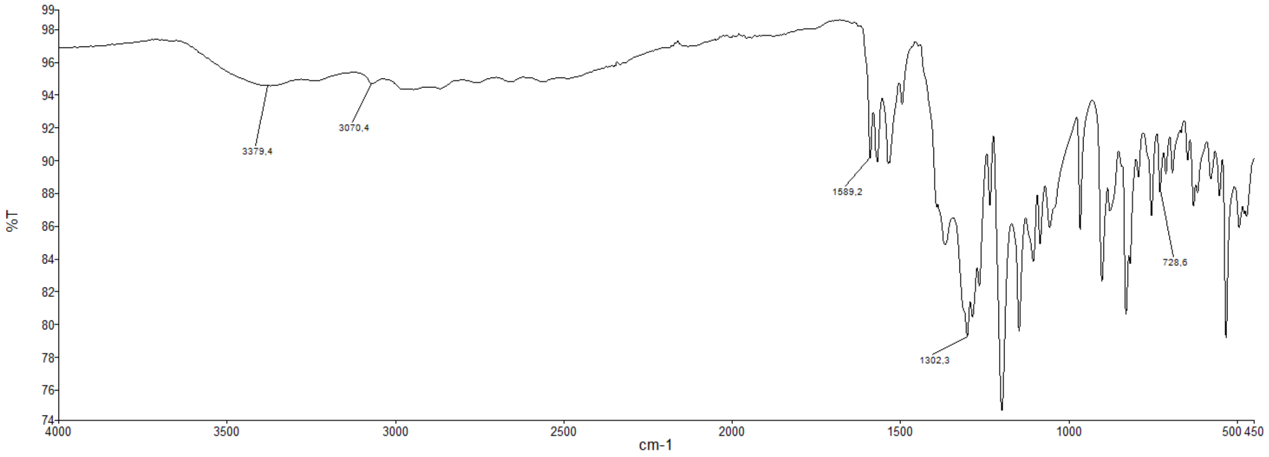


IR (ATR, cm$^{-1}$): 3379, 3070 w (O-H and C-H), 1589 m (C=N), 1302 s and 729 m (C=S).

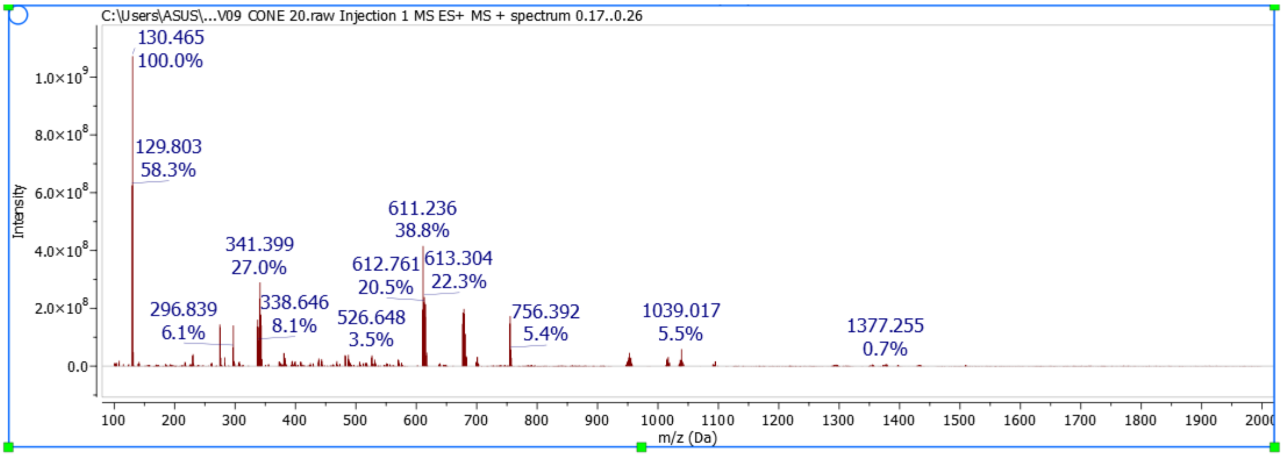


**CONE 20 V** ESI-MS *m*/*z* (%): 341.4 ([Ga+L-2H]$^{+}$, 27.0), 615.0 ([Ga+2L-2H]$^{+}$, 20.0), 678.8 ([Ga+2L+$NO_3^-$-H]$^{+}$, 18.4).

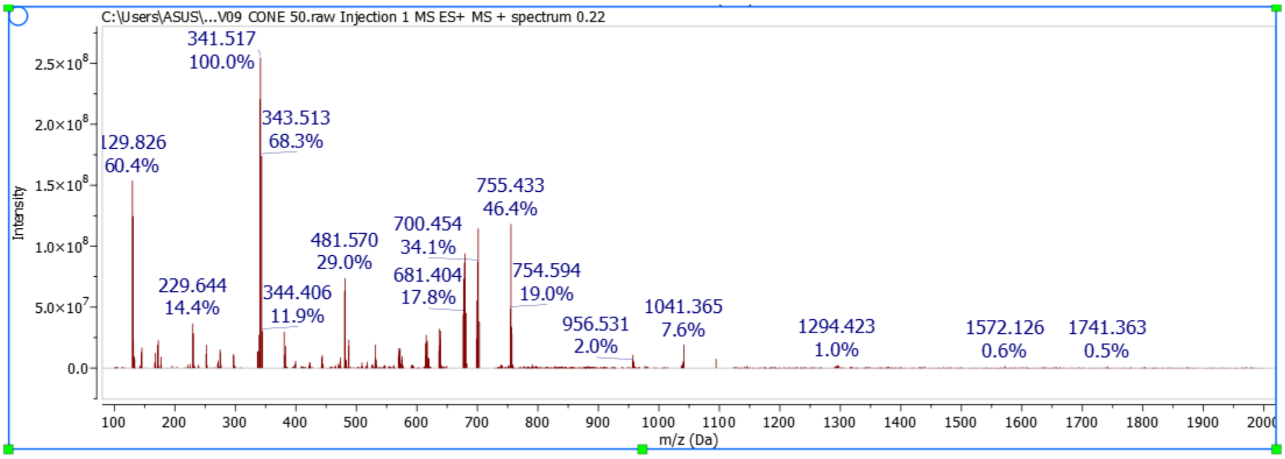


**CONE 50 V** ESI-MS *m*/*z* (%): 341.1 ([Ga+L-2H]$^+$, 100.0), 614.8 ([Ga+2L-2H]$^+$, 3.8), 679.0 ([Ga+2L+$NO_3^-$-H]$^+$, 38.8).

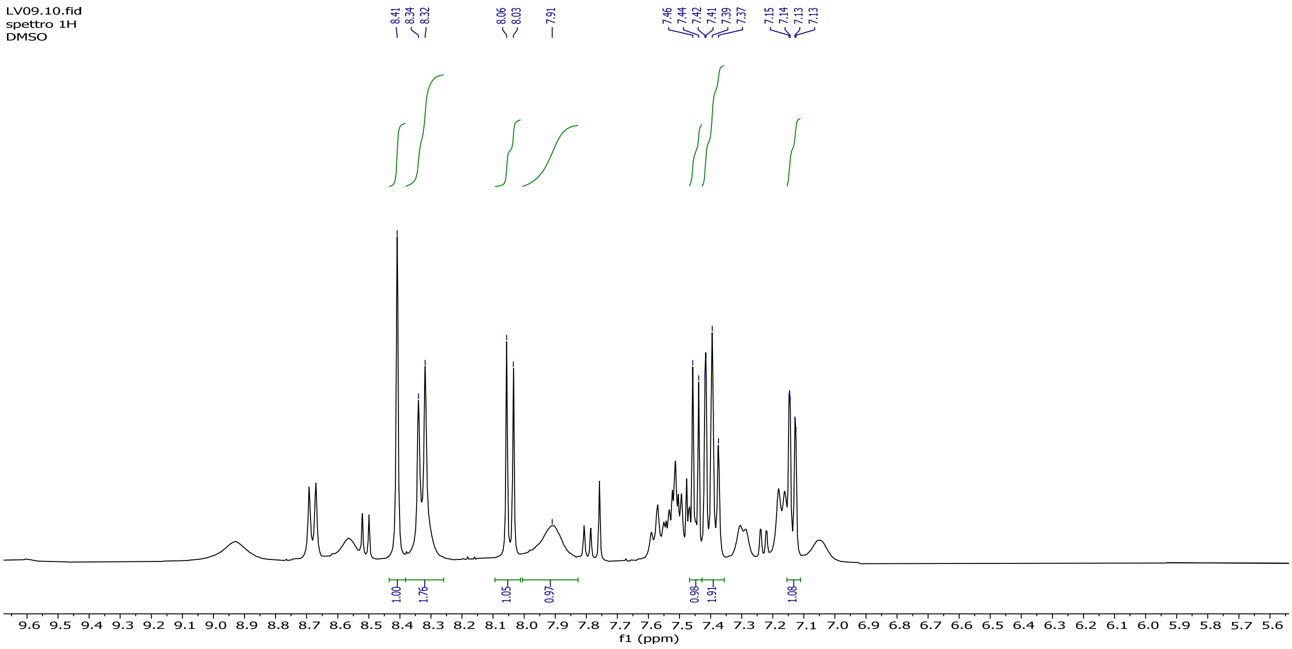


$^1$H-NMR (400 MHz, DMSO): [ppm] 8.40 (s, 1H, N=C-**H**), 8.33 (d, 1H, C**H** ar.), 8.04 (d, 1H, C**H** ar.), 7.91 (s, 1H, N-**H**), 7.45 (dd, 1H, C**H** ar.), 7.39 (t, 1H, C**H** ar.), 7.14 (dd, 1H, C**H** ar.).

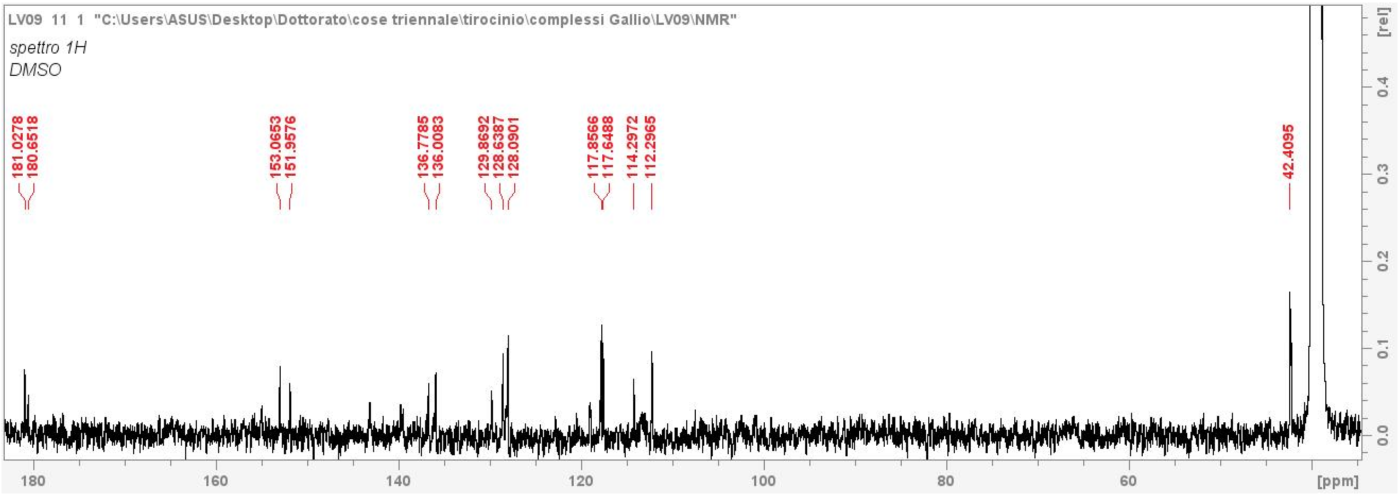


$^{13}$C-NMR (400 MHz, DMSO): [ppm] 181.0, 180.7 (**C**=N aliph.), 153.0 (**C**=S), 152.0 (**C**-OH), 143.2 (**C**=N ar.), 136.8, 136.0 (C=**C**=N ar.), 129.9, 128.6, 128.1 (C=**C**=C ar.), (C=**C**H-C ar., N-para), 119.1 (C=**C**H-C ar., OH-para), 117.9, 117.6 (C=**C**H-C ar., N-meta), 114.3 (C=**C**H-C ar., OH-para), 112.3 (C=**C**H-C ar., OH-ortho), 42.4 (**C**$H_3$).

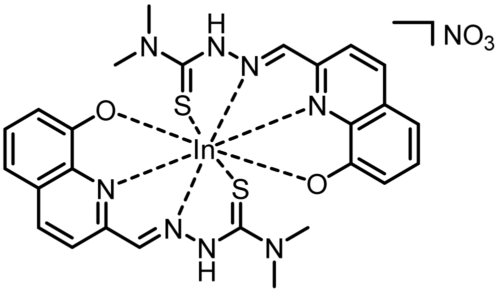


**In3**

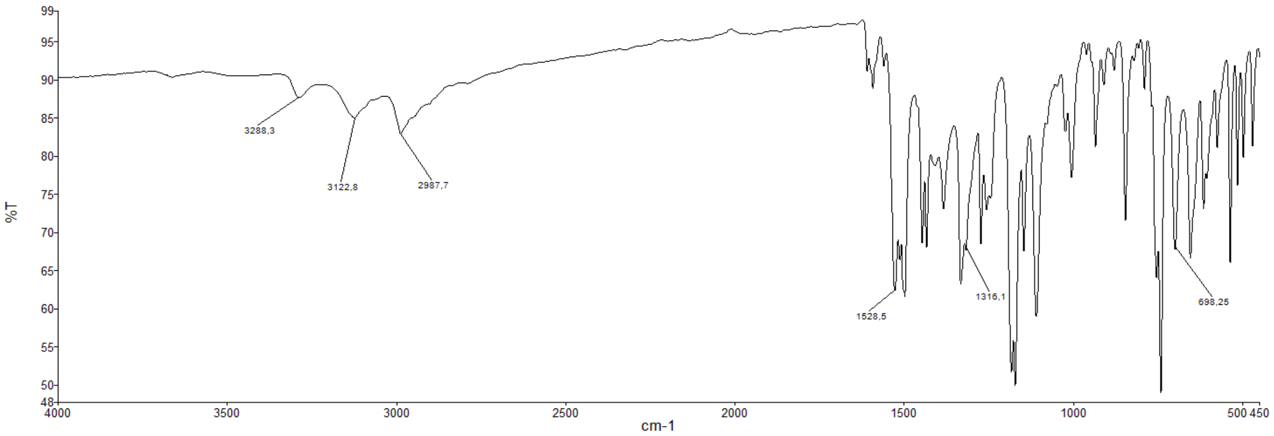


IR (ATR, cm[-1]): 3288, 3123 and 2988 w (N-H, O-H and C-H), 1529 m (C=N), 1316 and 698 m (C=S).

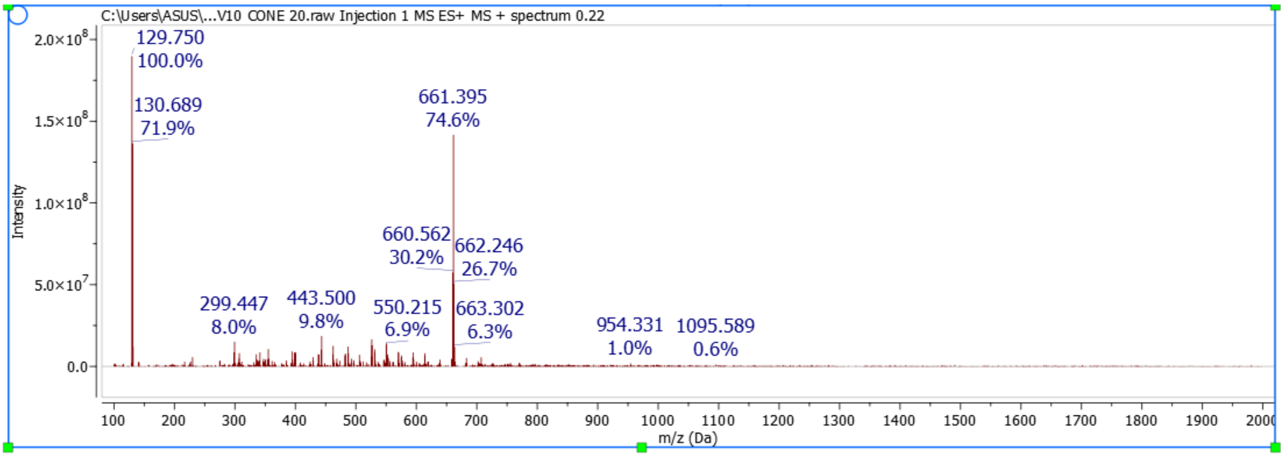


**CONE 20 V** ESI-MS *m*/*z* (%): 661.4 ([In+2L-2H]$^+$, 74.6).

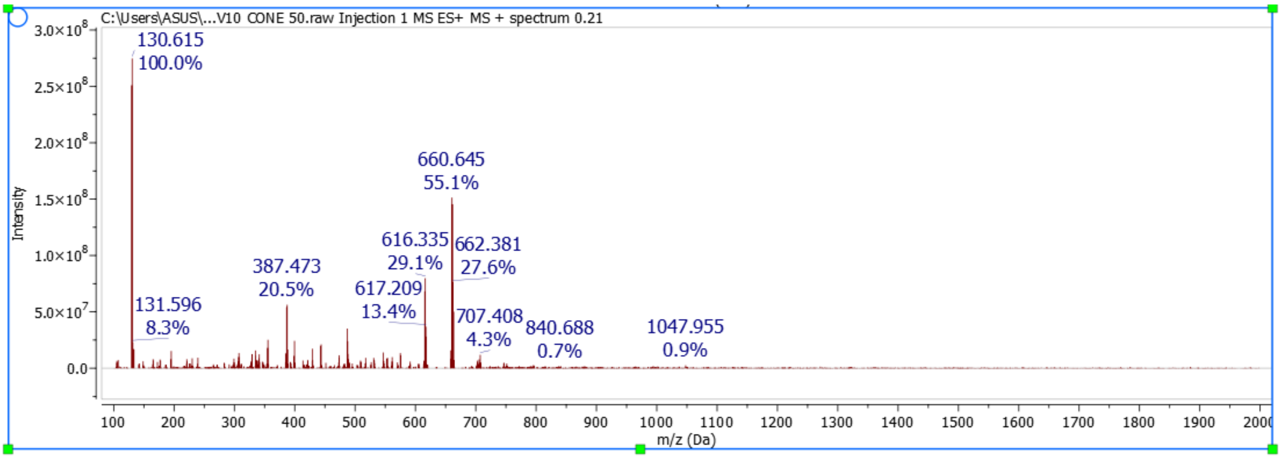


**CONE 50 V** ESI-MS *m*/*z* (%): 387.4 ([In+L-2H]$^+$, 20.5), 661.1 ([In+2L-2H]$^+$, 55.1).

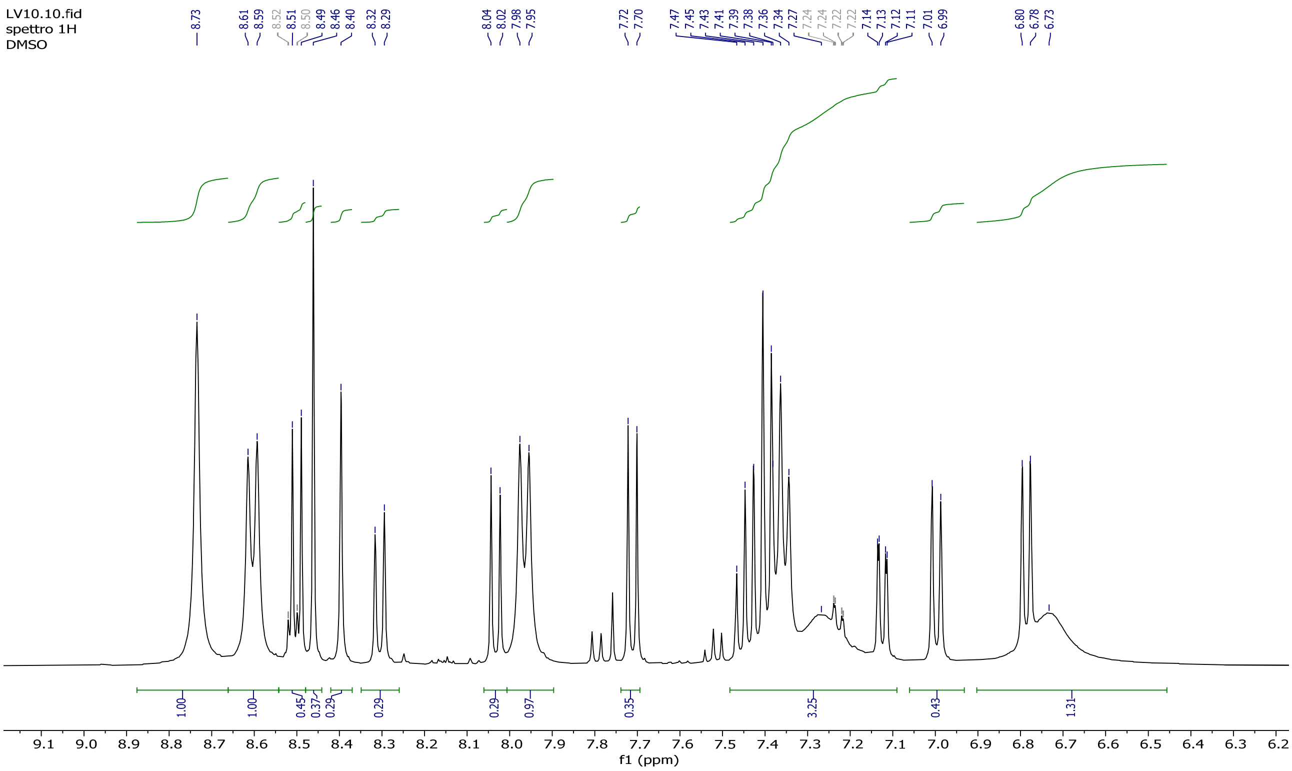


$^{1}$H-NMR (400 MHz, DMSO): [ppm] 8.75 (s, 1H, N=C-**H**), 8.60 (d, 1H, C**H** ar.), 7.95 (d, 1H, C**H** ar.), 7.40 (br, 1H, C**H** ar.), 7.40 (br, 1H, C**H** ar.), 7.27 (s, 1H, N**H**), 6.79 (br ,1H, C**H** ar.).

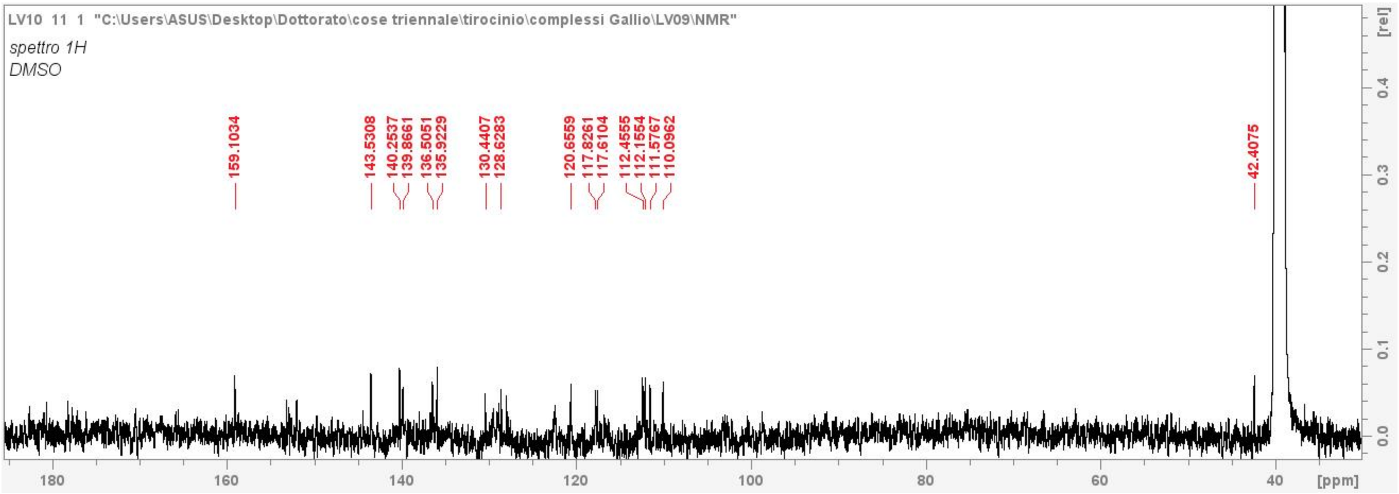


$^{13}$C-NMR (400 MHz, DMSO): [ppm] 180.7, 178.2 (**C**=N aliph.), 159.1 (**C**=S), 153.2 (**C**-OH), 152.0 (**C**=N ar.), 143.5, 140.3, 139.9 (C=**C**=N ar.), 136.5 (C=**C**=C ar.), 131.0 (C=**C**H-C ar., N-para), 128.6, 128.1 (C=**C**H-C ar., OH-para), 120.7 (C=**C**H-C ar., N-meta), 117.8, 117.6 (C=**C**H-C ar., OH-para), 112.5, 112.2, 111.6, 110.1 (C=**C**H-C ar., OH-ortho), 42.4 (**C**$H_3$).

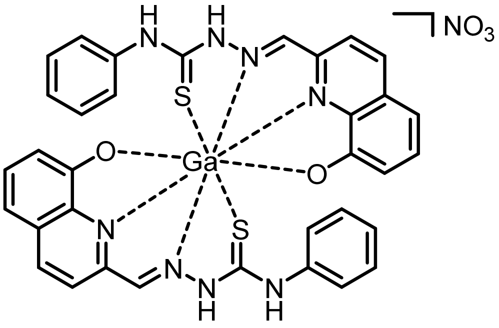

**Ga4**

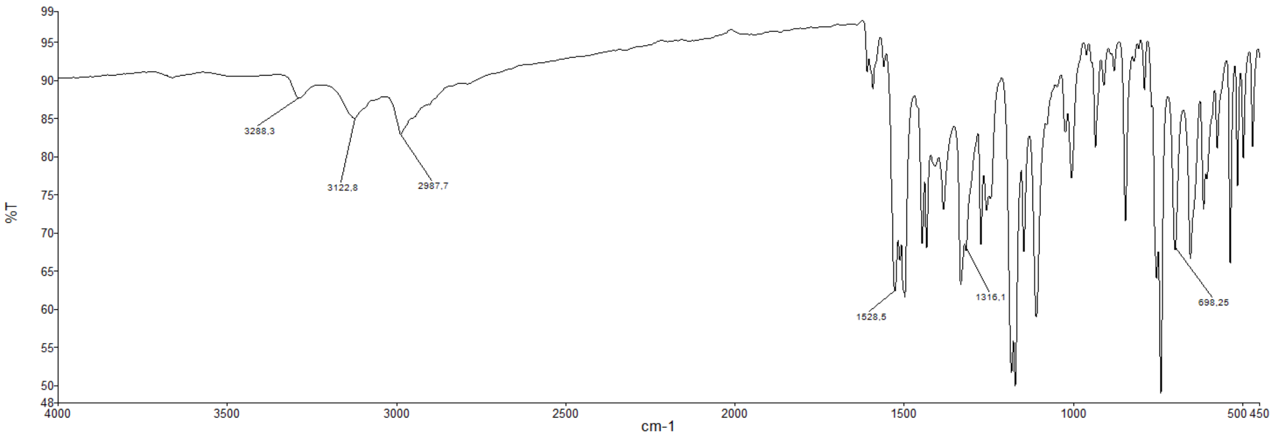


IR (ATR, cm$^{-1}$): 3288, 3123 and 2988 w (N-H, O-H and C-H), 1529 m (C=N), 1316 and 698 m (C=S).

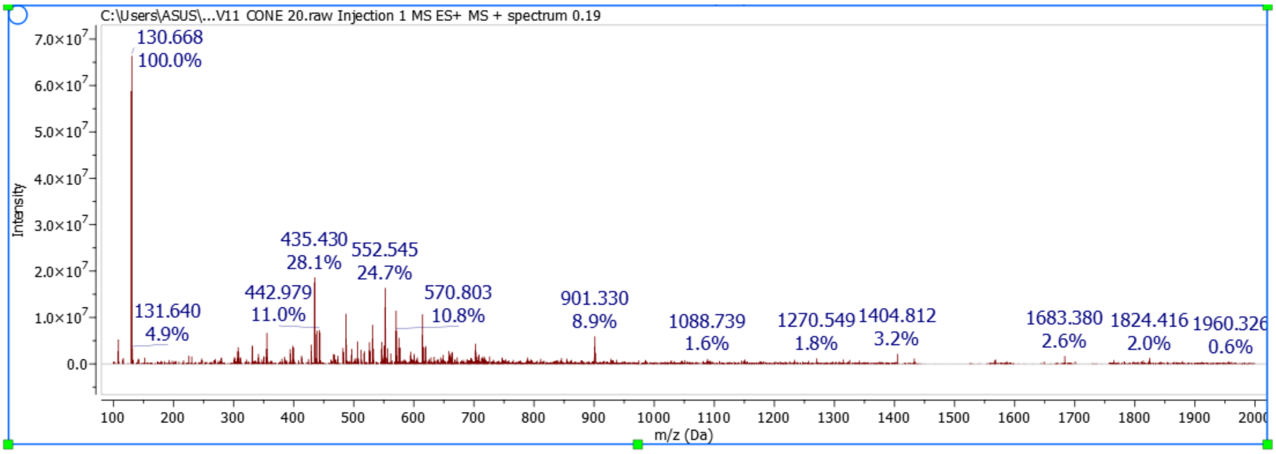


**CONE 20 V** ESI-MS *m*/*z* (%): 435.4 ([Ga+L+EtOH-2H]$^{+}$, 28.1).

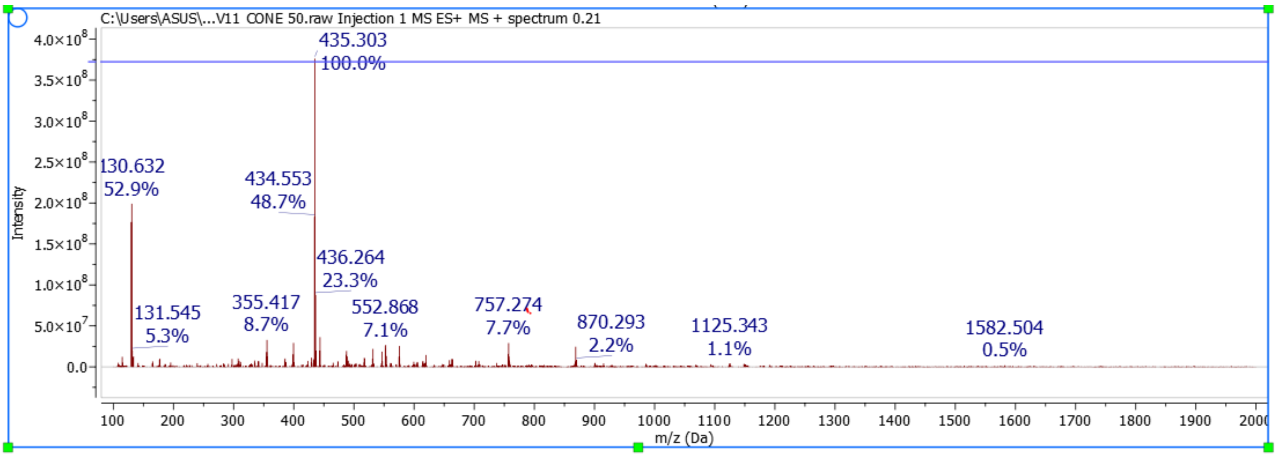


**CONE 50 V** ESI-MS *m*/*z* (%): 435.3 ([Ga+L+EtOH-2H]$^+$, 100.0), 757.3 ([Ga+2L+EtOH-2H]$^+$, 7.7).

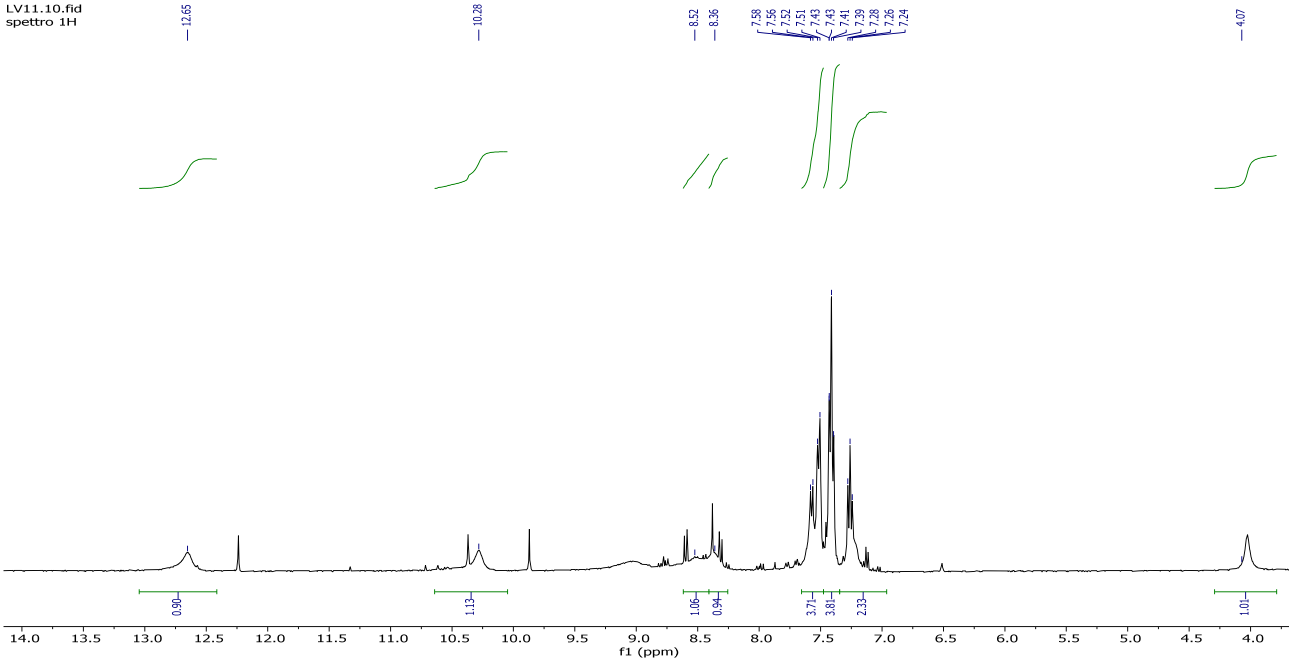


$^1$H-NMR (400 MHz, DMSO): [ppm] 12.65 (s, 2H, N**H**), 10.28 (s, 2H, N**H**), 8.52 (s, 1H, N=C-**H**), 8.36 (s, 1H, N=C-**H**), 7.58-7.24 (m, 20H, C-**H** ar.).

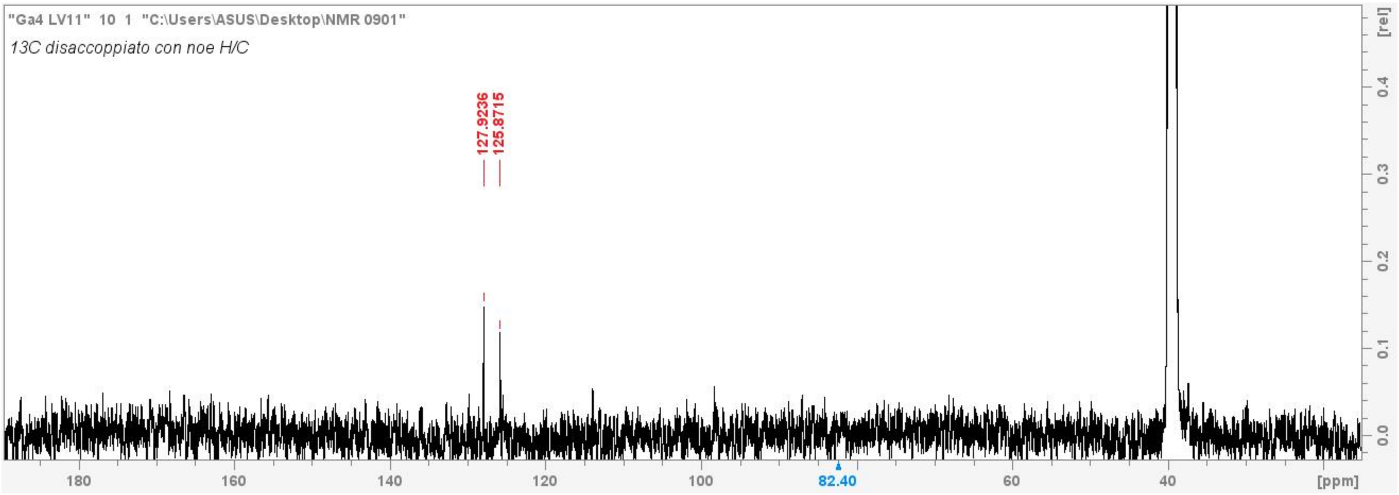


$^{13}$C-NMR (400 MHz, DMSO): [ppm] 177.0, 168.2 (**C**=N aliph.), 163.0, 155.6 (**C**=S), 143.2, 141.7 (**C**-OH), 139.1, 136.0, 132.7, 132.2, 129.9, 127.9, 125.9, 125.5, 120.21, 119.9, 113.9, 113.0, 109.5, 108.0, 101.8, 100.8, 98.3 (**C** ar.).

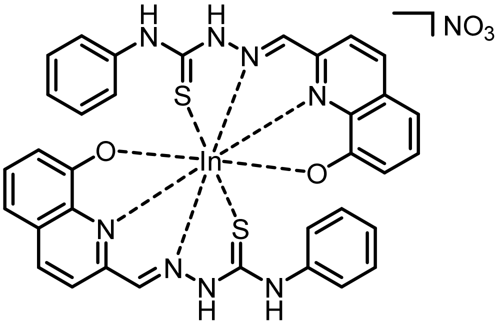

**In4**

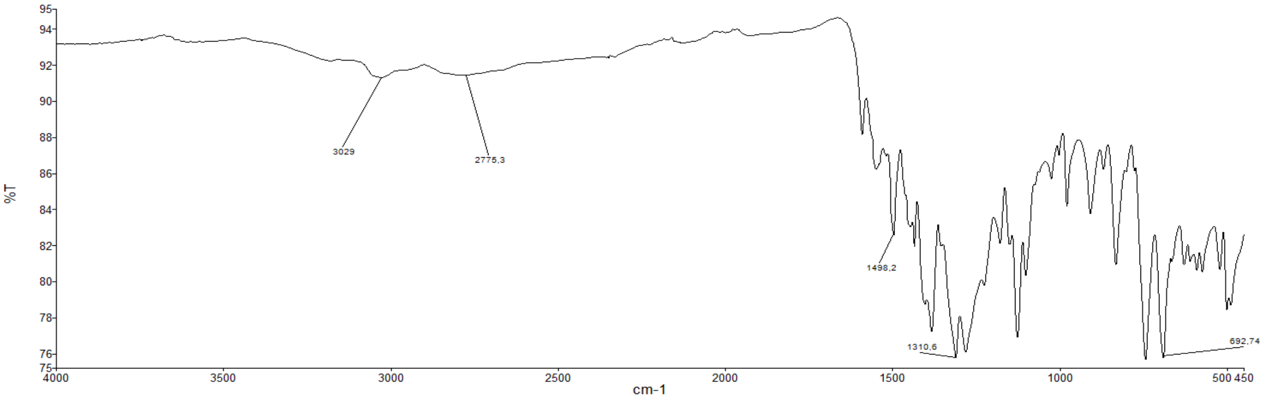


IR (ATR, cm$^{-1}$): 3029 and 2775 w (N-H, O-H and C-H), 1498 m (C=N), 1311 and 693 s (C=S).

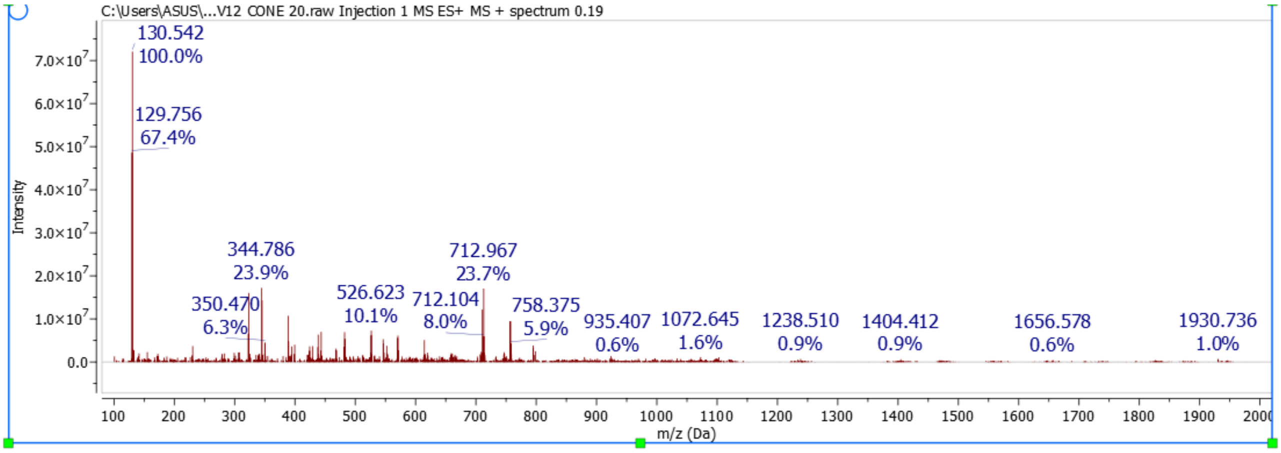


**CONE 20 V** ESI-MS *m/z* (%): 435.3 ([In+L-2H]$^+$, 4.6), 481.3 ([In+L+EtOH-2H]$^+$, 1.8), 757.3 ([In+2L-2H]$^+$, 15.6).

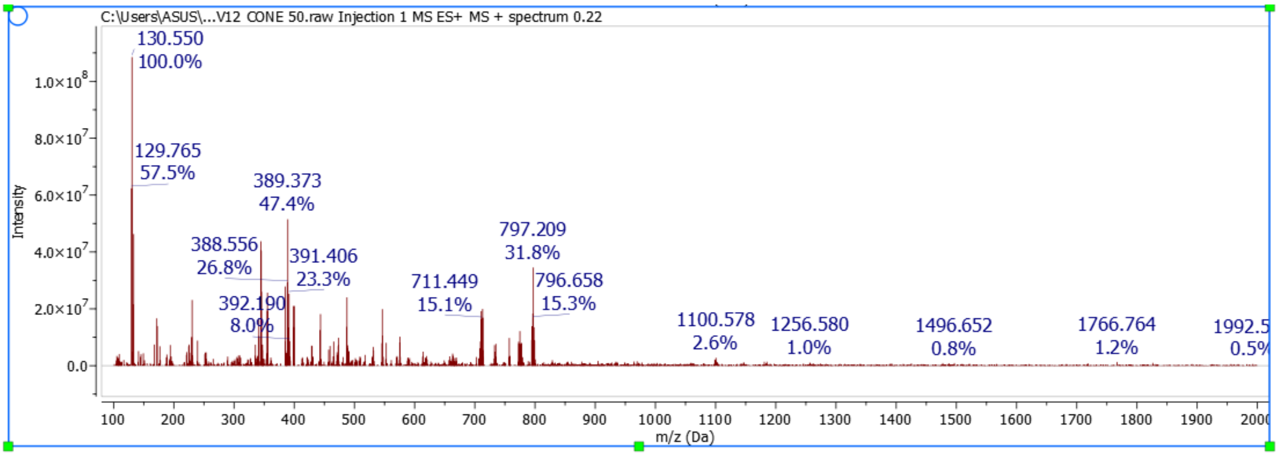


**CONE 50 V** ESI-MS *m/z* (%): 757.0 ([In+2L-2H]$^+$, 8.4).

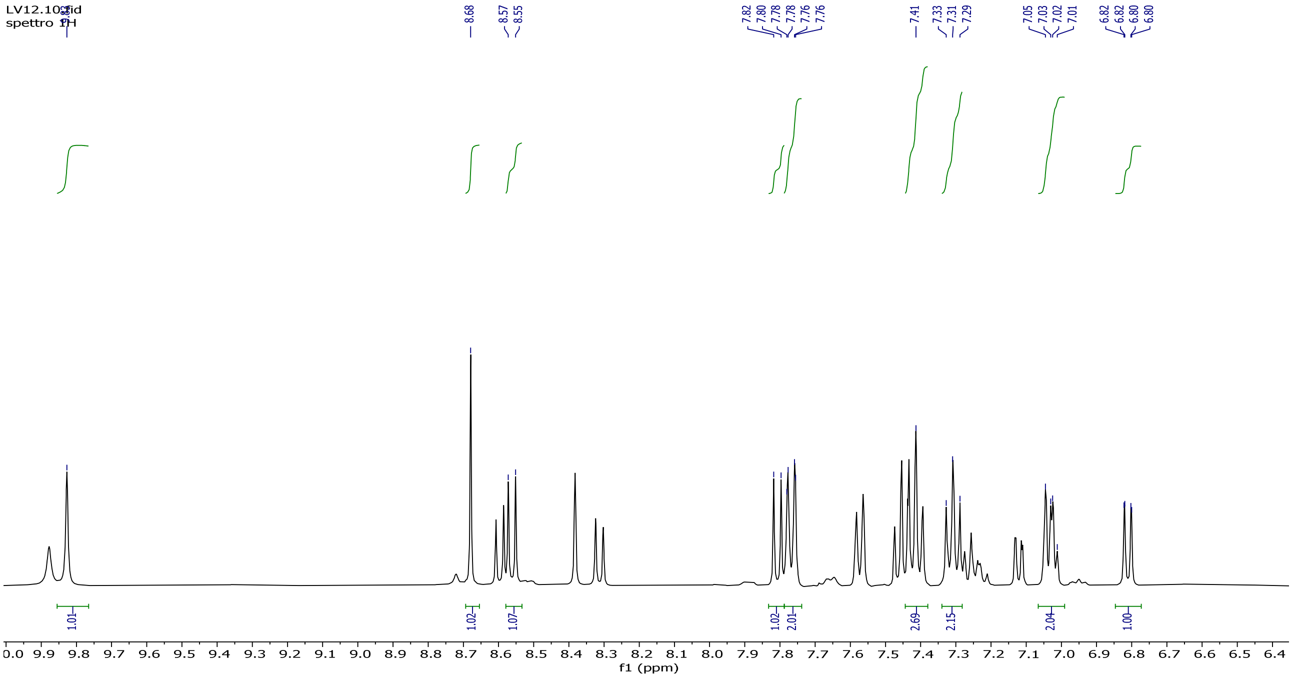

$^{1}$H-NMR (400 MHz, DMSO): [ppm] 9.83 (s, 1H, N**H**), 8.68 (s, 1H, N=C**H**), 8.56 (d, 1H, C**H** ar.), 7.81 (d, 1H C**H** ar.), 7.78 (dd, 2H, C**H** ar.), 7.41 (t, 1H, C**H** ar.), 7.31 (t, 2H, C**H** ar.), 7.04 (dd, 1H, C**H** ar.), 7.03 (t, 1H, C**H** ar.), 6.81 (dd, 1H, C**H** ar.).

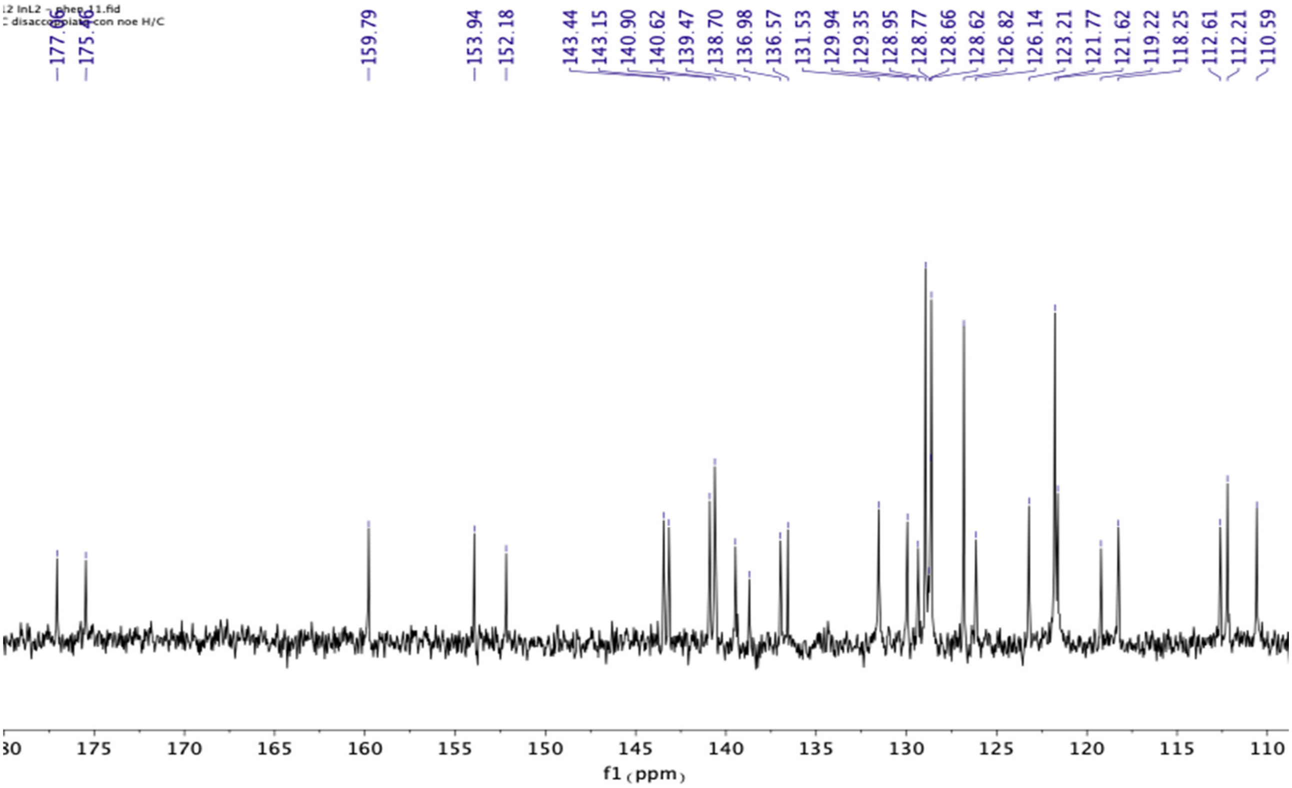


$^{13}$C-NMR (400 MHz, DMSO): [ppm] 177.1, 175.5 (**C**=N aliph.), 159.8, 153.9 (**C**=S), 152.2, 143.4 (**C**-OH), 143.2, 140.9, 140.6, 139.5, 138.7, 137.0, 136.6, 131.5, 129.9, 129.4, 129.0, 128.8, 128.7, 126.8, 126.1, 123.2, 121.8, 121.6, 119.2, 118.3, 112.6, 112.2, 110.6 (**C** ar.).
